# Supplementary figures and images for: Anthropogenic impacts on tidal creek sedimentation since 1900
Source: PLoS One. 2023 Jan 18;18(1):e0280490. doi: 10.1371/journal.pone.0280490 (PMC9847910; doi:10.1371/journal.pone.0280490)

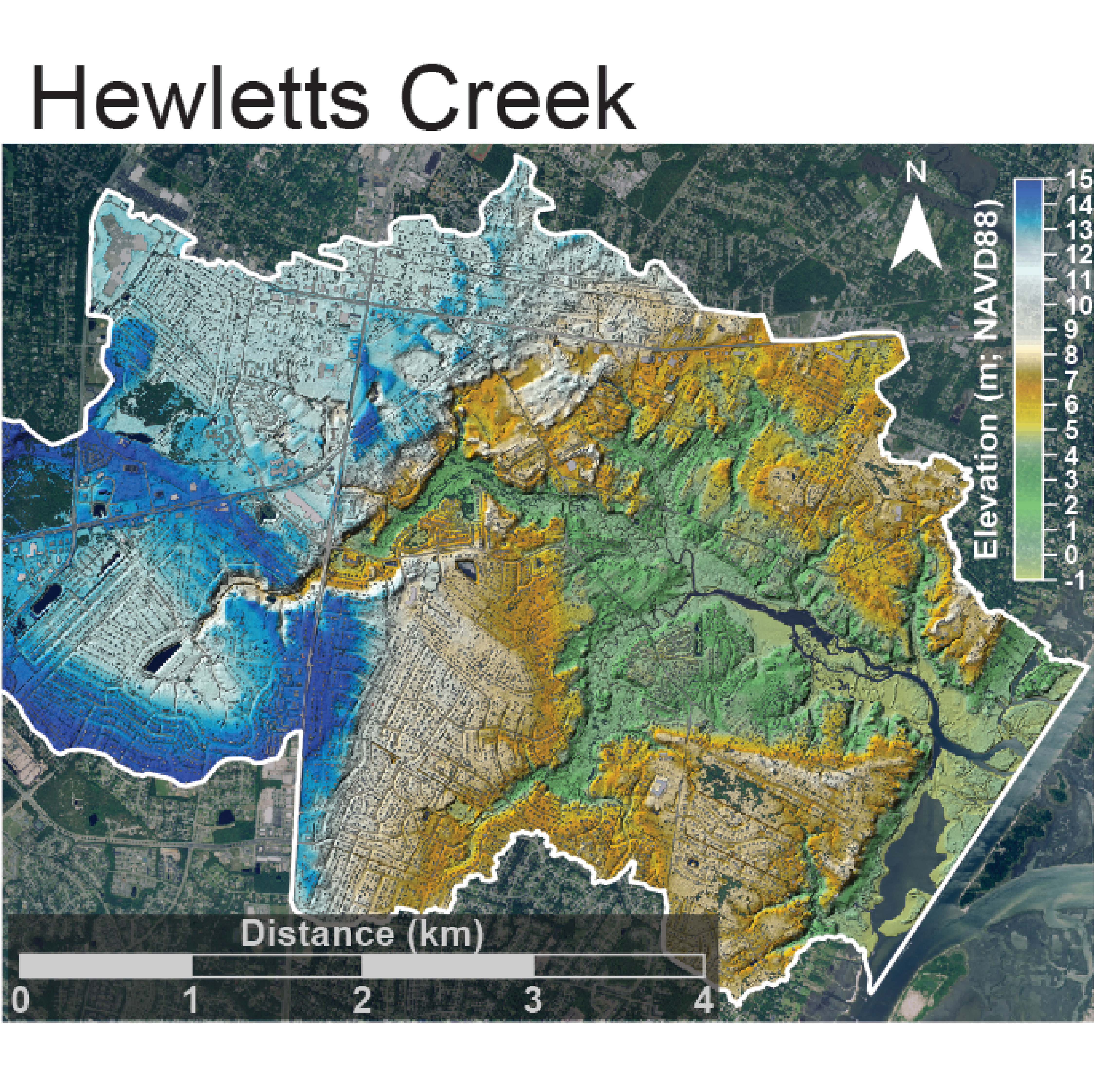

Supplement: S2 Fig — Digital elevation model of Hewletts Creek (Site 11) highlighting the Carolina Bay with a central retention pond in the northwestern part of the watershed. (TIF) [file pone.0280490.s002.tif]
