# Supplementary material for: Anthropogenic impacts on tidal creek sedimentation since 1900
Source: PLoS One. 2023 Jan 18;18(1):e0280490. doi: 10.1371/journal.pone.0280490 (PMC9847910; doi:10.1371/journal.pone.0280490)
Supplement: Attachment — Submitted filename: PONE-D-22-15917 with comments.pdf [file pone.0280490.s004.pdf]

PLOS ONE

Anthropogenic impacts on tidal creek sedimentation since 1900

--Manuscript Draft--

**Manuscript Number:**	PONE-D-22-15917
**Article Type:**	Research Article
**Full Title:**	Anthropogenic impacts on tidal creek sedimentation since 1900
**Short Title:**	Anthropogenic impacts on tidal creek sedimentation since 1900
**Corresponding Author:**	Molly Bost, Ph.D.<br>University of North Carolina at Chapel Hill Institute of Marine Sciences<br>Atlantic Beach, NC UNITED STATES
**Keywords:**	Sedimentation<br>210-Pb<br>Coastal land-use change<br>Tidal Creek
**Abstract:**	Land cover and use around the margins of estuaries has shifted since 1950 at many sites in North America due to development pressures from higher population densities. Small coastal watersheds (tidal creeks) are ubiquitous along estuarine margins and most of this coastal land-cover change occurred in tidal creek watersheds. A change in land cover could modify the contribution of sediments from tidal creek watersheds to downstream areas and affect estuarine habitats that rely on sediments to persist or are adversely impacted by sediment loading. The resilience of wetlands to accelerating relative sea-level rise depends, in part, on the supply of lithogenic sediment to support accretion and maintain elevation; however, subtidal habitats such as oyster reefs and seagrass beds are stressed under conditions of high turbidity and sedimentation. Here we compare sediment accumulation rates before and after 1950 using 210 Pb in 12 tidal creeks across two distinct regions in North Carolina, one region of low relief tidal-creek watersheds where land cover change since 1959 was dominated by fluctuations in forest, silviculture, and agriculture, and another region of relatively high relief tidal-creek watersheds where land-use change was dominated by increasing suburban development. At eight of the creeks, mass accumulation rates (g cm -2 y -1) measured at the outlet of the creeks increased contemporaneously with the largest shift in land cover, within the resolution of the land-cover data set (~5-years). All but two creek sites experienced a doubling or more in sediment accumulation rates (cm yr -1) after 1950 and most sites experienced sediment accumulation rates that exceeded the rate of local relative sea-level rise, suggesting that there is an excess of sediment being delivered to these tidal creeks and that they may slowly be infilling. After 1950, land cover at one site changed little, as did mass accumulation rates, and another site did not record an increase in mass accumulation rates at the creek outlet when a massive increase in development occurred that included the construction of retention ponds. These abundant tidal-creek watersheds have little relief, area, and flow, but they are impacted by changes in land cover more, in terms of percent area, than their larger riverine counterparts, and down-stream areas are highly connected to their associated watersheds. This work expands the scientific understanding of connectivity between lower coastal plain watersheds and estuaries, and provides important information for coastal zone managers seeking to balance development pressures and environmental protections.
**Order of Authors:**	Molly C. Bost, Ph.D.
	Charles D. Deaton, M.S.
	Antonio B. Rodriguez, PhD
	Brent A. McKee, PhD
	F. Joel Fodrie, PhD
	Carson B. Miller, M.S.
**Additional Information:**	

Powered by Editorial Manager® and ProduXion Manager® from Aries Systems Corporation

Question	Response
**Financial Disclosure**<br><br>Enter a financial disclosure statement that describes the sources of funding for the work included in this submission. Review the submission guidelines for detailed requirements. View published research articles from *PLOS ONE* for specific examples.<br><br>This statement is required for submission and **will appear in the published article** if the submission is accepted. Please make sure it is accurate.<br><br>**Unfunded studies**<br>Enter: *The author(s) received no specific funding for this work.*<br><br>**Funded studies**<br>Enter a statement with the following details:<br>• Initials of the authors who received each award<br>• Grant numbers awarded to each author<br>• The full name of each funder<br>• URL of each funder website<br>• Did the sponsors or funders play any role in the study design, data collection and analysis, decision to publish, or preparation of the manuscript?<br>• **NO** - Include this sentence at the end of your statement: *The funders had no role in study design, data collection and analysis, decision to publish, or preparation of the manuscript.*<br>• **YES** - Specify the role(s) played.<br><br>* typeset	FJF and ABR<br>2016-H-056<br>NC Division of Marine Fisheries<br>https://deq.nc.gov/about/divisions/marine-fisheries/grant-programs/coastal-recreational-fishing-license-grant-program#funding-information<br><br>Funders had no role in study design, data collection and analysis, decision to publish, or preparation of the manuscript
**Competing Interests**<br><br>Use the instructions below to enter a competing interest statement for this submission. On behalf of all authors, disclose any competing interests that could be perceived to bias this work—acknowledging all financial support and any other relevant financial or non-financial competing interests.	The authors have declared that no competing interests exist.

Powered by Editorial Manager® and ProduXion Manager® from Aries Systems Corporation

This statement is required for submission and **will appear in the published article** if the submission is accepted. Please make sure it is accurate and that any funding sources listed in your Funding Information later in the submission form are also declared in your Financial Disclosure statement.<br><br>View published research articles from *PLOS ONE* for specific examples.<br><br>**NO authors have competing interests**<br><br>Enter: *The authors have declared that no competing interests exist.*<br><br>**Authors with competing interests**<br><br>Enter competing interest details beginning with this statement:<br><br>*I have read the journal's policy and the authors of this manuscript have the following competing interests: [insert competing interests here]*<br><br>* typeset	
**Ethics Statement**<br><br>Enter an ethics statement for this submission. This statement is required if the study involved:<br><br>• Human participants<br>• Human specimens or tissue<br>• Vertebrate animals or cephalopods<br>• Vertebrate embryos or tissues<br>• Field research<br><br>Write "N/A" if the submission does not require an ethics statement.<br><br>General guidance is provided below. Consult the submission guidelines for detailed instructions. **Make sure that all information entered here is included in the Methods section of the manuscript.**	N/A

Powered by Editorial Manager® and ProduXion Manager® from Aries Systems Corporation

**Format for specific study types**<br><br>**Human Subject Research (involving human participants and/or tissue)**<br>• Give the name of the institutional review board or ethics committee that approved the study<br>• Include the approval number and/or a statement indicating approval of this research<br>• Indicate the form of consent obtained (written/oral) or the reason that consent was not obtained (e.g. the data were analyzed anonymously)<br><br>**Animal Research (involving vertebrate animals, embryos or tissues)**<br>• Provide the name of the Institutional Animal Care and Use Committee (IACUC) or other relevant ethics board that reviewed the study protocol, and indicate whether they approved this research or granted a formal waiver of ethical approval<br>• Include an approval number if one was obtained<br>• If the study involved *non-human primates*, add *additional details* about animal welfare and steps taken to ameliorate suffering<br>• If anesthesia, euthanasia, or any kind of animal sacrifice is part of the study, include briefly which substances and/or methods were applied<br><br>**Field Research**<br><br>Include the following details if this study involves the collection of plant, animal, or other materials from a natural setting:<br>• Field permit number<br>• Name of the institution or relevant body that granted permission	
**Data Availability**<br><br>Authors are required to make all data underlying the findings described fully available, without restriction, and from the time of publication. PLOS allows rare exceptions to address legal and ethical concerns. See the PLOS Data Policy and FAQ for detailed information.	Yes - all data are fully available without restriction

Powered by Editorial Manager® and ProduXion Manager® from Aries Systems Corporation

A Data Availability Statement describing where the data can be found is required at submission. Your answers to this question constitute the Data Availability Statement and **will be published in the article**, if accepted.<br><br>**Important:** Stating 'data available on request from the author' is not sufficient. If your data are only available upon request, select 'No' for the first question and explain your exceptional situation in the text box.<br><br>Do the authors confirm that all data underlying the findings described in their manuscript are fully available without restriction?	
**Describe where the data may be found in full sentences. If you are copying our sample text, replace any instances of XXX with the appropriate details.**<br><br>• If the data are **held or will be held in a public repository**, include URLs, accession numbers or DOIs. If this information will only be available after acceptance, indicate this by ticking the box below. For example: *All XXX files are available from the XXX database (accession number(s) XXX, XXX.).*<br>• If the data are all contained **within the manuscript and/or Supporting Information files**, enter the following: *All relevant data are within the manuscript and its Supporting Information files.*<br>• If neither of these applies but you are able to provide **details of access elsewhere**, with or without limitations, please do so. For example:<br><br>*Data cannot be shared publicly because of [XXX]. Data are available from the XXX Institutional Data Access / Ethics Committee (contact via XXX) for researchers who meet the criteria for access to confidential data.*<br><br>*The data underlying the results presented in the study are available*	All relevant data are within the manuscript and its Supporting Information files.

from (include the name of the third party and contact information or URL).<br>• This text is appropriate if the data are owned by a third party and authors do not have permission to share the data.<br><br>* typeset	
Additional data availability information:	Tick here if the URLs/accession numbers/DOIs will be available only after acceptance of the manuscript for publication so that we can ensure their inclusion before publication.

Powered by Editorial Manager® and ProduXion Manager® from Aries Systems Corporation

Manuscript

Click here to access/download;Manuscript;Tidal Creeks_PLOSOne_2022.docx

Anthropogenic impacts on tidal creek sedimentation since 1900

[1]Molly C. Bost, [1]Charles D. Deaton, [1,2]Antonio B. Rodriguez, [2]Brent A. McKee, [1,2]F. Joel Fodrie, [1]Carson, B. Miller

1. *Institute of Marine Sciences, University of North Carolina at Chapel Hill, 3431 Arendell St., Morehead City, NC 28557, USA.*
2. *Department of Earth, Marine and Environmental Sciences, University of North Carolina at Chapel Hill, Chapel Hill, NC 27599-3300, USA.*

Keywords: ^{210}Pb, tidal creek, coastal, land-use change, sedimentation

Abstract

Land cover and use around the margins of estuaries has shifted since 1950 at many sites in North America due to development pressures from higher population densities. Small coastal watersheds (tidal creeks) are ubiquitous along estuarine margins and most of this coastal land-cover change occurred in tidal creek watersheds. A change in land cover could modify the contribution of sediments from tidal creek watersheds to downstream areas and affect estuarine habitats that rely on sediments to persist or are adversely impacted by sediment loading. The resilience of wetlands to accelerating relative sea-level rise depends, in part, on the supply of lithogenic sediment to support accretion and maintain elevation; however, subtidal habitats such as oyster reefs and seagrass beds are stressed under conditions of high turbidity and sedimentation. Here we compare sediment accumulation rates before and after 1950 using ^{210}Pb in 12 tidal creeks across two distinct regions in North Carolina, one region of low relief tidal-creek watersheds where land cover change since 1959 was dominated by fluctuations in forest, silviculture, and agriculture, and another region of relatively high relief tidal-creek watersheds where land-use change was dominated by increasing suburban development. At eight of the creeks, mass accumulation rates (g cm^{-2} y^{-1}) measured at the outlet of the creeks increased contemporaneously with the largest shift in land cover, within the resolution of the land-cover data set (~5-years). All but two creek sites experienced a doubling or more in sediment accumulation rates (cm yr^{-1}) after 1950 and most sites experienced sediment accumulation rates that exceeded the rate of local relative sea-level rise, suggesting that there is an excess of sediment being delivered to these tidal creeks and that they may slowly be infilling. After 1950, land cover at one site changed little, as did mass accumulation rates, and another site did not record an increase in mass accumulation rates at the creek outlet when a massive increase in

development occurred that included the construction of retention ponds. These abundant tidal-creek watersheds have little relief, area, and flow, but they are impacted by changes in land cover more, in terms of percent area, than their larger riverine counterparts, and down-stream areas are highly connected to their associated watersheds. This work expands the scientific understanding of connectivity between lower coastal plain watersheds and estuaries, and provides important information for coastal zone managers seeking to balance development pressures and environmental protections.

Introduction

Drowned river-mouth estuaries primarily receive sediment from their formative rivers. A portion of that sediment is deposited in the estuary and forms the substrate on which ecologically and economically important habitats, such as sea grass, oyster reefs, mangroves, and salt marshes, colonize. Changes in sediment accumulation in the coastal zone, however, can result in either the proliferation or demise of those habitats and the quantity and type of ecosystem services provided [1, 2]. A shift toward more frequent and/or larger pulses of sediment delivered to an estuary can force habitats to transition, such as tidal flats transitioning to intertidal saltmarsh, or sandy subtidal flats transitioning to muddy subtidal flats [3]. If sedimentation is too high, seagrass and oyster reefs can become buried and converted to mud or sandflat environments [4]. A reduction in sediment supply can cause intertidal habitats like saltmarsh and mangrove to drown with sea-level rise [5- 9] and communities subsequently lose the protection those habitats provide from storms [10- 13].

The inherent vulnerability of coastal areas to climate change, including increases in storminess and the rate of sea-level rise, is compounded with higher population densities and development pressures. Coastal county populations throughout the United States have increased by 39% since the 1970s and population density is over six times greater in coastal counties than inland ones [14]. Populations in many North American Atlantic, Gulf, and Pacific coastal counties that directly border an estuary have doubled since 1950 [15]. Additionally, coastal areas support tourism industries that host millions of visitors each year [16], suggesting that coastal development likely increases more than what the census population would suggest. Expanding coastal populations increase total risk to communities from large storms and sea-level rise; however, the associated changes in land cover also impact sediment delivery to depositional environments such as oyster reefs, salt marshes, mangroves, seagrasses, and tidal flats that provide communities some protection from shoreline erosion and flooding [17- 19].

Humans have significantly modified river sediment load, discharge, and the degree to which watersheds are connected to the coast [20, 21]. Land-cover changes, such as clearing forests, commonly promotes soil erosion and increases sediment load in rivers, with relief providing an important first-order control on the extent of landscape erosion and sediment transport [22]. Development of urban and suburban centers is associated with the channelization of catchment basins, increased flow velocities and increased connectivity between landscapes and downstream environments; however, reservoirs interrupt sediment transport across the coastal plain [23- 25]. An inferred decrease in sediment delivery to estuaries, based on river gauges positioned ~15-100 km landward of river outlets, has been attributed to the construction of dams, which peaked in the United States around 1950 [26, 27]. This inference assumes land-cover changes in the lower coastal plain contributes little to estuarine sedimentation, and conflict

with direct measures of increasing sediment accumulation rates in many North American estuaries [15] and a net global increase in deltaic land area over the past 30 years [28]. Coastal population has been rising for more than half a century and is a common proxy for land-use change [29]. Most of the population increase and associated change in land cover near estuaries occurs along the shoreline, seaward from where the main river discharges into the heads of estuaries, and those downstream areas are connected to estuaries via multiple smaller watersheds confined to the coastal zone. These smaller watersheds positioned along the margins of estuaries could be important sources of sediment to the coastal zone because larger rivers discharge into the heads of estuaries or directly into the open ocean.

Connectivity between upstream and downstream in a fluvial system generally decreases as catchment area increases because of sediment storage in floodplains, therefore, connectivity could be high in small coastal plain rivers with little to no floodplain. Higher vertical gradients are typically correlated with an increase in upstream to downstream connectivity, and low relief coastal plain rivers are assumed to be disconnected from land cover changes in their watershed [30]; however, there are examples where that assumption does not apply [3, 31, 19, 32, 33].

The objective of this study was to examine the effects of changes in land cover in small coastal zone watersheds, which vary by orders of magnitude in drainage basin size and relief, experience different tidal ranges, and have been impacted by different types of landscape change, on sediment accumulation in downstream environments. These small (<50 km^2) lower coastal-plain watersheds are abundant, the primary conduits of sediments and nutrients between uplands and coastal waters along the margins of estuaries and are commonly disregarded in estuarine sediment-flux models. While these coastal watersheds have little relief, area, and flow, they are more impacted by population growth and associated land-use change, in terms of percent

watershed area relative to watershed size. Unlike larger coastal plain and piedmont rivers, small coastal watersheds are not dammed and have no reservoirs to buffer the downstream estuary against land-use changes upstream. We mapped changes in land cover of small coastal-plain watersheds, identified periods of greatest change, and compared those periods to the depositional record since 1900 CE using the radiometric tracer ^{210}Pb. Linking changes in land cover to changes in sedimentation will provide guidance to managers and stakeholders on how to increase the resilience of coastal communities through wetland and soil conservation practices, which could include dredging, installation or removal of buffers along creeks, or modifications to existing or future developments (e.g., reductions in impervious surfaces).

Background

Tidal creek formation

Along passive continental margins, rivers incised valleys when lower sea-level exposed the break-in-slope between the low-gradient coastal plain and the higher gradient continental shelf [34, 35]. Smaller dendritic drainage networks also formed when sea level was lower on the surrounding landscapes of the lower coastal plain and subaerially-exposed inner shelf and carved smaller valleys into Pleistocene and older strata [36, 37]. These incised valleys and small drainage networks became estuaries and tidal creeks, respectively, as the landscape flooded during sea-level rise [38].

For this study, we define tidal creeks as small channel networks that drain lower-coastal plain watersheds less than 50 km^2, are tidal along their entire length, discharge into larger estuaries, lagoons, or back-barrier sounds, and are fringed by saltmarsh complexes along their

main stem. This definition excludes wetland channels [39], which can be dynamic over century timescales and lack an upland watershed. Tidal creeks developed within coastal prism and tributary incised valleys [37]. Coastal prism incised valleys formed across the break in slope between the low-gradient coastal plain and the steep shoreface of the previous highstand shoreline (Fig 1). Tributary incised valleys formed along the margins of larger incised trunk valleys. The high convexity along the edge of the incised valley, between the steep valley flank and the flat coastal plain, increased stream power and promoted erosion and knickpoint migration. The steep relic landscape morphology around estuarine areas when sea-level was lower is why tributary incised valley dimensions are commonly larger than what would be predicted by their small drainage basin size [37].

Fig 1. Study area map.
The topography and watershed boundaries of each tidal creek with the creek site numbers increasing from east to west (1-12). Six creeks were sampled in both New Hanover County (A), and Carteret County (B). A core from each creek was obtained from the same general location, with respect to the creek outlet, as shown for Ward Creek (C).

Tidal creeks are comprised of upper reaches where the channel is constricted and meandering along a fringing saltmarsh complex, and an embayed open-water region of confluence between the lower tidal-creek basin and the main estuary it drains into. In this open-water region of the tidal creek, salt marsh islands, oyster reefs, seagrass beds, and mudflats proliferate. Ensign et al. (2017) summarized marsh-mudflat morphodynamics in the embayed zone as being driven by waves, and marsh-channel morphodynamics in the meandering zone as being driven by tidal currents [40]. While the area of the embayed zone is covered by various amounts of structured subtidal and intertidal habitats, such as seagrass, oyster reef, and salt-marsh patches, the mouth of the meandering channel is always fringed by wetlands and commonly saltmarsh.

Tidal creek sedimentation

Sediment is supplied to a tidal creek from its drainage basin and from the estuary it drains into. Sediment moves primarily from upstream to downstream during rain events and sources include runoff, resuspension, and bank erosion. Tidal-creek watersheds have little relief, highly variable vegetation cover, and a wide range of anthropogenic impacts so the potential for erosion within the watershed can vary substantially across small spatial scales. Sediment moves from the estuary into the tidal creek during flood tide or storm surge and is sourced mainly from surrounding drainage basins, offshore, resuspension, and shoreline erosion.

Sediment deposition in the embayed zone of a tidal creek is driven by sediment supply and accommodation, which change over a variety of timescales. Accommodation is the amount of space available for sediments to accumulate in, which near the coast, is closely tied to the level of wave base and the rate of relative sea-level rise (RSLR; [41]). Over centennial timescales accommodation is mainly driven by RSLR and sediment flux by climate. Shorter timescales (annual to decadal) encompass changes in watershed land cover and storminess, which results in higher sediment flux to the tidal-creek embayment, and higher resuspension of the bed as wave base deepens, respectively. The water depth of the lower embayed portion of tidal creeks is not necessarily in equilibrium with storm wave base, as is common for larger estuaries and lagoons [42]. The tidal creek embayment within many tributary incised valleys has a surplus of sediment accommodation because they are oversized in comparison to the creek's watershed [37]. Tidal creeks in coastal prism incised valleys, however, are more accommodation

limited because the size of the lower embayed portion scales with the size of the tidal-creek watershed and contains numerous intertidal patches of oyster reefs and salt marshes.

In North Carolina, along the 180-km long coastline between Cape Lookout and Cape Fear there are four rivers that discharge into the heads of estuaries (North, Newport, White Oak, and New), but > 60 tidal creeks positioned seaward of bayhead deltas, 8-30 km from the ocean shoreline. The small size of tidal creek watersheds may be disproportionate to their importance as conduits for sediment transport from landscapes to estuaries and the coastal ocean because they are abundant, located in areas of expanding populations and creek discharge is strongly impacted by storminess.

Methods

Site Selection

The North Carolina coast (NC, U.S.A.) hosts extensive estuarine systems, a broad coastal plain, and recently modified coastal watersheds making it an ideal study area. Glacial eustatic fluctuations formed terraces and scarps along the lower coastal plain of NC [43]. The Suffolk paleoshoreline formed ~80 ka [44] at an elevation between 6 and 14 m, parallels the NC ocean shoreline from the southwest to Morehead City, NC, where its trend abruptly changes to the North forming a right angle or paleo cape [45]. The paleo cape at Morehead City divides the study area into two distinct zones. North of the paleo cape, the coastal plain seaward of the Suffolk Shoreline is characterized by a low slope, elevation <2 m, numerous tributary incised valleys [44], and an astronomical tidal range of 0.95 m at NOAA Station ID 8656483 near

Morehead City that decreases to about 0.37 m near Cedar Island, NC in southern Pamlico sound (NOAA Station ID 8655151). Southwest of the paleo cape is characterized by relatively steep coastal plain slopes with Carolina Bays typically positioned landward of the Suffolk Shoreline, numerous coastal prism incised valleys, and a tidal range of 1.21 m at NOAA Station ID 8658163. Carolina Bays are oval shaped depressions (<3 m deep) with elevated rims that vary in size (~100m – 4 km along the long axis) along the southeastern Atlantic coast and are oriented NW to SE [46]. Our study includes 12 tidal creeks in southeastern NC, split between Carteret County (CC; lower tidal range) and New Hanover County (NHC; higher tidal range; Fig 1). Of those 12 tidal creeks seven are within coastal prism incised valleys, six in NHC and one in CC (Gales Creek, Site 6), and five are within tributary incised valleys, all in CC (Fig 1).

Land-use change and digital elevation models

Watersheds were delineated by hand in ESRI ArcGIS using digital elevation models (DEMs) from lidar data collected in 2014 for CC and 2015 for NHC (± 0.12 m vertical; [47, 48]; Fig 1A and B).Watershed relief was calculated as the difference between the highest and lowest 10% of elevation points of the DEM (Table 1, Figs 1A and 1B).

Year	1	2	3	4	5	6	7	8	9	10	11	12
1959												
1964												
1969												
1970												
1975												
1982												
1993												
1996												
2001												
2006												
2010												
2016												

Table 1. Land-cover datasets.
The years land-cover classes were mapped in each tidal creek watershed (Green) with missing land cover data in orange. See Fig 1 for creek numbering scheme.

Land cover of each watershed area was classified at least every decade between the 1959 – 2016 study period (Table 2). From 1959-1993, land cover was digitized from georeferenced aerial imagery from the United States Geological Survey (USGS) Aerial Photo Single Frames records collection and National High-Altitude Photography (NHAP) program. Classifications used were forest, cleared forest, agriculture, developed, or water/intertidal. Changes between cleared forest and forest classifications were associated with silviculture operations. Land-cover from 1996-2016 was obtained from the Coastal Change Analysis Program (C-CAP; [49]) and reclassified to match the same categories as the earlier time-steps. Some of the aerial imagery data sets did not capture every watershed, which resulted in land cover being classified 11 times for nine tidal creeks and 10 times for three tidal creeks (Table 2).

Site ID	Creek Name	Dbs (km^2)	Watershed Relief (m)	Core Date	Core latitude (degrees)	Core longitude (degrees)	Approximate water depth (m)	Creek Valley Type
**1**	Oyster	11.76	2.30	2016-08-05	34.8270	-76.4623	1.1	Tributary
**2**	Tusk	1.88	2.68	2016-08-09	34.7463	-76.5139	1.2	Tributary
**3**	Sleepy	5.38	3.73	2016-07-21	34.7329	-76.5272	0.8	Tributary
**4**	Ward	14.96	2.44	2016-07-07	34.7907	-76.5658	1.1	Tributary
**5**	Ware	1.54	2.49	2016-08-24	34.7750	-76.6741	0.8	Tributary
**6**	Gales	7.78	9.48	2016-07-28	34.7321	-76.9075	1.2	Coastal Prism
**7**	Futch	15.44	11.88	2016-05-10	34.3041	-77.7532	0.75	Coastal Prism
**8**	Pages	20.35	14.60	2016-10-04	34.2816	-77.7786	1.1	Coastal Prism
**9**	Howe	14.24	13.47	2016-10-05	34.2559	-77.8065	1.1	Coastal Prism
**10**	Bradley	18.67	12.57	2016-09-20	34.2179	-77.8395	1	Coastal Prism
**11**	Hewletts	30.23	15.08	2016-09-12	34.8270	-76.4623	1.5	Coastal Prism
**12**	Whiskey	8.49	8.74	2016-09-12	34.1600	-77.8626	1.1	Coastal Prism

Table 2. Watershed characteristics and core locations.
Creek name with corresponding site number, Dbs=Drainage basin size or watershed area, watershed relief, core collection date and location, and creek type (Tributary or Coastal Prism incised valley).

The percent watershed area of each land cover class changes over time; however, there were typically two classes that changed the most over the 50-year period, with one class being replaced by another. We defined the major land-cover change (MLCC) for a watershed as the class with the highest percent contribution to the total change in land cover over the 50-year period. For every watershed, we defined the time boundary between pre- and post-MLCC as the midpoint between the dates of two land cover classification maps where a ≥15% change occurred within the record over ~10-year period (S1 Fig). The 15% change criterion is the 90th percentile of all the changes recorded between successive land cover classification maps for the MLCCs.

S1 Fig. Land-cover datasets since 1959.
Land cover classes shown as percent area of tidal creek watershed for each site in Carteret County (A) and New Hanover County (B). Land cover was classified at least once per decade. Bars connecting land cover datasets indicate MLCC for each creek watershed.

Field sampling and radiometric dating

To maximize the potential for obtaining a complete high-resolution sedimentary record, each tidal creek was sampled where the channel widened and transitioned from wetland-dominated to embayed or within the bayhead shoreline ([50]; Fig 1C). A push core (1 m) was collected in 2016 from each creek bottom (water depth ~1m; Table 2) with the aid of a sledgehammer. Sediment cores were transported back to the laboratory in a vertical position, extruded into 1-cm intervals (~10 cm^3) and frozen. Samples were then weighed, freeze-dried, and weighed again to calculate porosity. Dry bulk density values were calculated assuming quartz composition. Disaggregated subsamples were used to measure percent organic matter by loss on ignition (LOI; [51]) and grain size (<2000 to 0.04 μm fraction) using a Cilas laser particle size analyzer. The remaining samples were then sorted through a 63-micrometer sieve for radioisotope analysis.

The fine-grained fraction (<63 microns) was used for isotope-dilution alpha spectrometry to quantify ^{210}Pb via the granddaughter isotope, ^{210}Po, which is assumed to be in secular equilibrium with ^{210}Pb [52- 55]. Raw ^{210}Pb data and dry bulk density for each depth interval for all 12 creek cores, both of which are needed for geochronology modelling, are presented in the attached S1 File. To obtain accumulation rates, ^{210}Pb dating methods including the Constant Flux and Constant Flux Constant Sedimentation models described in Sanchez-Cabeza and Ruiz-Fernandez (2012) were used. For this study, the Constant Flux model (CF; widely known as the Constant Rate of Supply model; [56, 57]) was of the most interest because it assumes that ^{210}Pb flux to the sediment surface is constant and the initial concentrations and the mass accumulation

rates (MAR) of individual layers may change, but they must be inversely proportional. The CF model, therefore, is useful for tracking sedimentation rates that vary over time. Depth integrated ^{210}Pb inventories were calculated for each sediment core and used to calculate sediment accumulation rates (SAR) with the CF model. This model provides ages and accumulation rates for discrete intervals within the core allowing for direct comparison with the land-cover time-series data. All reported p-values are the result of t-tests assuming unequal variances between sample groupings.

S1 File. **Raw 210-Pb data.**

Results

Drainage basin size and relief

The drainage basins of the tidal creeks vary in area and relief by an order of magnitude. Coastal prism drainage basins are generally larger and have greater relief than the tidal creeks within tributary incised valleys (Table 1) with Hewletts Creek (Site 11) having the largest area (30.23 km^2) and relief (15.08 m), Ware Creek (Site 5) having the smallest area (1.54 km^2), and Oyster Creek (Site 1) having the lowest relief (2.3 m). The drainage basin size of tidal creeks increases with relief and drainage basin size is a power function of relief except for two outliers, Oyster (Site 1) and Ward (Site 4) creeks (Fig 2). Both outliers are tributary incised valleys with larger drainage basins than what would be predicted by their low reliefs. Ward Creek is positioned along the flank of the North River incised valley, which is 8-10 m deep at the confluence [37]. When sea level was lower and the area was exposed, the relic steep slope and

high relief along the flank of the North River incised valley promoted incision, knickpoint migration, and expansion of the Ward Creek drainage basin above what would be predicted from watershed relief [37]. The other tidal creeks that formed within tributary incised valleys merge with their associated larger river systems on the inner continental shelf, farther seaward than where Ward Creek merges with the North River [44], and the size of those drainage basins, at the present headwater locations, is controlled mainly by relief. The Oyster Creek outlier has the lowest watershed relief and a disproportionately large drainage basin area that has been highly modified by humans. Extensive ditching of nearby farmland and wetlands and creation of a 1.25 km^2 waterfowl impoundment site has increased watershed area above its natural state.

Fig 2. Drainage basin size vs relief.
Tidal creek drainage basin size increases exponentially with relief. Tidal creeks are enumerated as shown on Fig 1. Oyster Creek (1) and Ward Creek (4) are outliers excluded from the regression.

Land cover 1959 to 2016

Land cover of all 12 tidal creek watersheds was dominated by forest, cleared forest, and agriculture in the 1959 imagery (Fig 3A). During the next 57 years, development of the creek watersheds in NHC increased to an average of 50.2 ±5.5% of total watershed area, while the CC creek watersheds remained dominated by forest, cleared forest, and agriculture (Fig 3B). The creek watersheds in NHC are adjacent to the expanding Wilmington, NC urban center and developed into suburban landscapes, while the creek watersheds in CC remained relatively rural over the same period. Development of the NHC creek watersheds, the MLCC class, increased the most, ~20-26%, between the early 1970s and middle 1990s (S1 Fig). Initiation of MLCC generally occurred first in the watersheds closer to Wilmington, NC; ~1964 for Whiskey Creek

in the west and ~1989 for Futch Creek in the east. In the tidal creek watersheds of CC, the MLCC class was cleared forest except for Ward Creek (Site 4) where the MLCC class was agriculture (S1 Fig). The initiation of MLCC (cleared forest) of the Oyster Creek watershed (Site 1) occurred between 1982 and 1993. The percent area of forest and cleared forest fluctuated after 1975 in the Tusk Creek (Site 2) and Sleepy Creek (Site 3) watersheds, mainly the result of silviculture operations, with the start of MLCC occurring between 1975 and 1982 with 18% and 28% increase in cleared forest at each respective site. Agriculture area rapidly increased between 1975 and 1982 in the Ward Creek watershed (Site 4). Land cover of the Ware Creek watershed (Site 5) changed the least throughout the period in all classes and never exceeded the 15% threshold for the initiation of MLCC, with a maximum increase of only 11% cleared forest between 1993 and 1996. The land cover of the Gales Creek watershed (Site 6) increased 15% in developed area between 1964 and 1982, the highest increase in development of all the CC sites, and forest and cleared forest area fluctuated after 1964. The MLCC class in Gales Creek, however, was cleared forest which decreased 38.4% in area between 1964 and 1975, which corresponded to at 35% gain in forest area (S1 Fig). Increasing forested area is an unlikely mechanism for mobilizing sediment. Despite the decrease in cleared forest area fitting our criteria for the MLCC class, the smaller contemporaneous increase in development is more relevant for impacting sedimentation downstream.

Fig 3. Earliest land-cover and most recent land-cover.
Tidal-creek watershed percent land cover of each class in 1959 and 2016 (A). Agriculture was the MLCC for Oyster Creek (B) and development was the MLCC for Howe Creek (C).

Tidal creek sediment composition

Changes in sediment composition and texture with depth in a sediment core can indicate changes in sediment source or support a hypothesized change in sedimentary regime. Most of the tidal creek cores showed minor variation in sediment composition with depth, but there were some differences in texture between CC and NHC. The CC creek sites generally had less sand than the NHC creek sites. Distinct depositional environments over which the watersheds formed, capacity for transporting sand, and sediment texture of adjacent estuarine source areas explains the broad differences in sediment texture between counties. The tidal creek watersheds in NHC are positioned on the sandy relatively high-relief Suffolk Scarp paleoshoreline, while most of the tidal creek watersheds in CC formed on old inner-continental shelf muddy substrate with low relief [44]. The Gales Creek watershed in CC (Site 6) drains a low relief area of the Suffolk Scarp and the core lacks sand. Importantly, the embayments of the NHC creeks are positioned closer to modern sandy tidal deltas and have a greater tidal range and capacity for transporting sand than CC creeks. The one exception is Oyster Creek (Site 1) that had between 37- 55% sand (Fig 4) throughout the sediment core. Of the CC creek sites, Oyster creek has the most direct connection to sandy Core Sound [58]. There was also relatively little change in percent organic matter with depth for most of the cores (Fig 4). The top ~3 cm of the cores from the NHC sites had a higher % organic matter than what was measured below, likely due to incomplete biodegradation of surface organic carbon. Hewletts Creek (Site 11) showed some variation in organic matter and sediment composition downcore, which could be attributed to a change in sediment regime (Fig 4).

Fig 4. Changes in the composition of each core with depth.
Cumulative percent of clay (black), silt (dark grey), and sand (light grey) and percent organic matter (while circles) are shown for each 1-cm thick core sample. Mid-point depths are plotted from the top of the bed (0 cm) to the base of the ^{210}Pb profile.

Mass accumulation rates through time

Long term average (1900-2016) MARs were higher in tidal creeks within coastal prism incised valleys than tributary incised valleys (0.21 ± 0.03 g cm^2 yr^{-1} and 0.12 ± 0.03 g cm^2 yr^{-1}, respectively, P=0.033) excluding Bradley Creek (Site 10) which was mixed throughout the profile making accumulation rates impossible to derive. Average MAR from 1900-1950 was the same in both creek valley types, but from 1950-2016, average MAR was higher in creeks within coastal prism incised valleys than tributary incised valleys (0.22 ± 0.004 and 0.14 ± 0.002 g cm^2 yr^{-1}, respectively, P= 0.032). Most creeks showed an increasing trend in MARs from 1900 to 2016 and most of the increase occurred in the second half of the century (1950-2016), the only exception being Ward Creek (Site 4) with consistently low MARs (Fig 5A).

Fig 5. MAR since 1900.
Mass accumulation rates (MAR) for cores from tidal creeks within tributary incised valleys (A) and coastal prism incised valleys (B). Bradley Creek could not be analyzed due to mixing. Vertical errors are measurement uncertainty representative of alpha spectrometry and horizontal error shows the amount of time each 1-cm slice represents in the core.

The MARs of Oyster, Tusk, Sleepy, Gales, Futch, Pages, Hewett's, and Whiskey creeks (Sites 1-3, 6, 7, 8, 11, and 12) rapidly increased following the mid-1970s or early 1980s, reached a maximum in the 1990s or early 2000s and subsequently stabilized or decreased. In contrast, the

MARs at Howe (Site 9) and Ware (Site 5) creeks increased until ~1960, the beginning of our land-cover time-series data set, but then showed small fluctuations until 2016.

Discussion

The MARs of the tidal creeks were higher at sites within coastal prism incised valleys than tributary incised valleys (Figs 5 and 6). The difference in MARs between valley types could, in part, be a result of accommodation differences between coastal prism and tributary incised valleys. Tributary incised valley systems have an oversized embayed area for sediments to accumulate in. Any commensurate increase in sediment flux would result in deposition of a thinner layer and lower recorded accumulation rates in tributary incised valleys than coastal prism incised valleys. In addition, tidal creeks within coastal prism incised valleys have a greater percent of intertidal area and associated oyster reefs than tidal creeks within tributary incised valleys, and oysters promote deposition through filter feeding and the production of feces and pseudofeces.

The MARs of 8 of the 12 tidal creeks increased during the period of the land cover record (1959-2016). Constraining the relative proportions of the various sediment sources that contributed to the increase at each creek is not possible with our data set; however, given that we included numerous tidal creeks in the study, many of which are in proximity to each other, it is possible to identify likely forcing mechanisms. The cores collected for this study were all from tidal creek outlets or bayhead shorelines [50] where an increase in MAR eventually forms tidal flats that become colonized with salt marsh as the creek outlet moves basin-ward. At the bayhead shoreline of tidal creeks, an increase in accumulation rate of the bed must be associated with an

increase in sediment load and/or a decrease in the depth of bed erosion. Tidal creeks receive some sediment from larger rivers through their connection with estuaries and the coastal ocean and from shoreline erosion. If an increase in sediment supply to those connected water bodies caused the increase in MAR measured at most of our sites, then the timing of the increase should correspond with the sediment loading of those larger rivers and the increase recorded as a synchronous shift in depositional regime of many adjacent tidal creeks. There is no hydrological connectivity between the NHC tidal creek sites and the nearby Cape Fear River, but sediment loading in the Neuse and the Newport rivers could have influenced sedimentation in the CC tidal creeks. The timing of the increase in MAR for the tidal creeks in CC, however, occurred after 1975 CE, postdating the increase in sediment loading of the Newport River (1964; [3]) and the Neuse River (~1940; [59, 15]; Fig 6).

Fig 6. MAR since 1950 vs land-cover change.
MAR (cyan) and tidal creek watershed land cover since 1950 as seen in S1 Fig. Tributary incised valleys (A) and Coastal Prism incised valleys (B) with MLCC as bold bar between the two land-cover time steps with >15% change. The earlier part of the sedimentary record is shown in Figure 5.

Coastal rain events and flooding from storm surge disproportionately affect tidal creeks as compared to large river systems due to their low elevation, small relief, and position along the shoreline. Changes in coastal storminess can promote shoreline erosion and increase sediment supply; however, it would also increase the depth of erosion at the creek outlet and increase subtidal area. Furthermore, the proximity of the sites to each other suggests that a change in storminess would also result in a simultaneous change in MAR, but the timing of the increase differs among creek sites. An increase in storms with wind > 55.5 km h^{-1} (primarily northeasters) did occur around 2000 in the area near the CC sites [60] but increased storminess postdates the

diachronous increase in MAR among creeks (Fig 5). Transport of sediment by sheet flow in tidal creek watersheds occurs during storms and coastal NC has entered a wetter climatic regime where storm events are bringing more extreme precipitation patterns [61, 62]. The predicted increase in storm intensity and frequency in the region [63], should result in further increased connectivity between tidal creek watersheds and downstream depositional environments.

Relating changes in MAR with changes in land cover using quantitative methods is problematic because it would require us to coarsen the temporal resolution of the accumulation rates to match the decadal land cover record, removing clear trends in accumulation rates that exist at the sub-decadal time scale (Fig 6). The timing of the increase in MAR after 1959 corresponded with the initiation of MLCC at eight of the creek sites (Sites 1-3, 6-8, 11, and 12), cleared forest for the creek sites within tributary incised valleys and suburban development for creek sites within coastal prism incised valleys (Fig 6). Sites 1-3 experienced variations in cleared-forest area from silviculture activities that started in the mid-1970s through the mid-1980s. Immediately after the initiation of silviculture, MAR accelerated. The creek sites impacted primarily by changes in silviculture recorded a more persistent acceleration in MAR as forested and cleared forest areas transition back and forth with reestablishment and harvesting of pine trees. The land cover of the Gales Creek watershed (Site 6) experienced the largest change between 1964 and 1975 with a decrease in cleared forest area and an increase in developed area. These land-cover changes corresponded with accelerating MARs between 1964 and 1975 and a transition from silviculture to development and agriculture in the watershed. The MARs of sites 7, 8, and 12 (Futch, Pages, and Whiskey creeks, respectively) accelerated rapidly during periods of increasing development in the late-1980s, mid-1970s, and mid-1960s, respectively.

Development of the Hewletts Creek (Site 11) watershed initiated in the 1950s, but the increase in MAR was not recorded until ~1980. The pattern of development across the Hewletts Creek watershed progressed from landward to seaward, initiating in the upper part of the watershed within a Carolina Bay depression (~3.5 km^2 within a 30.2 km^2 watershed; S2 Fig). This depression, with a central man-made retention pond, likely acted as a depocenter for ~11% of the total Hewletts Creek watershed, limiting drainage to the lower watershed and creek. The increase in development prior to 1982 occurred mostly within the confines of the Carolina Bay depression and was not recorded as an increase in MAR because the depression disconnected the modified landscape from the creek. The increase in development between 1982 and 1993 occurred outside of the Carolina Bay depression throughout the lower watershed and MAR accelerated rapidly during the same period. The core from Hewletts Creek also showed a decrease in organic matter and an increase in sand between 1982 and 1996. Only Hewletts Creek recorded changes in sediment composition as MAR accelerated and unlike the other sites, most of the increase in development occurred on the sandy Suffolk Scarp, which makes up 46% of the 30.23 km^2 watershed.

S2 Fig. Carolina Bay within Site 11.
Digital elevation model of Hewletts Creek (Site 11) highlighting the Carolina Bay with a central retention pond in the northwestern part of the watershed.

The creek sites that recorded an increase in MAR with large changes in developed land cover (Futch, Pages, Hewletts, and Whiskey; Sites 7, 8, 11, and 12) show a subsequent levelling off or decrease in MAR during the most recent part of the sedimentary record. Maintenance of constant or decreasing MARs occurred in the absence of any large changes in land cover of the creek watersheds. It is likely that surface sediment was eroded from the watershed and

transported to the creeks during the construction phase of development, which included clearcutting forest, and once developed there was little sediment available that could be mobilized and exported to the tidal creeks.

Tidal creeks 4, 5, and 9 experienced changes in watershed land cover with no commensurate increase in MAR at the outlet. The NE trend of Ward Creek (Site 4) makes it susceptible to resuspension and flushing during SW wind events, the prevailing wind direction in the area. The Ward Creek sedimentary record consistently recorded the lowest MARs of all sites and unsupported ^{210}Pb was only measured down to 7 cm in the core, indicating the orientation of the creek is conducive to sediment bypass near the bay head shoreline, not deposition. Large changes in land cover of the Ward Creek watershed post MLCC in 1982, such as the increase in cleared forest from 6-24% from 1996-2001, likely did promote an increase in sediment flux to coastal areas; however, the core was not obtained from an efficient depocenter where that would be recorded. Ware Creek (Site 5) experienced little change in land cover since 1959, always below the 15% threshold defining the initiation of a MLCC, but preserved some variability in sediment accumulation rates, most within error, which aligned with the timing of small changes in land-cover (Fig 6). The Howe Creek (Site 9) sedimentary record also preserved little changes in MAR since 1959; however, the development area of the watershed more than doubled between 1982 and 1996. Unlike the other creeks in NHC that showed an increase in MAR as developed area of the watershed increased, the Howe Creek watershed had a relatively large area dedicated to retention ponds, >2% of the watershed as compared to <0.5% in other creeks. The retention ponds functioned to interrupt runoff from reaching downstream areas and likely restricted sediment transport following land-cover changes in the Howe Creek watershed.

The presence of structured habitats in tidal creeks and their connected estuaries are required for healthy and productive ecosystems. Saltmarsh and mangrove habitats rely on sedimentation to maintain position in the tidal frame with accelerating RSLR; however, sediment loading can also have negative effects on ecosystems. Harmful heavy metals can sorb onto fine-grained particles and poor water quality from sediment loading commonly results in restrictions on commercial fishing, smothering of benthic flora and fauna, and lowered primary productivity from increased light attenuation [64-66]. The two tidal creeks with high-resolution sedimentation records that did not record an increase in MAR near the bay head shoreline (Sites 5 and 9) provide guidance on possible strategies for mitigating sediment loading. Land cover change of the Ware Creek watershed impacted <11% of the area and no corresponding increase in MAR was recorded indicating that small-scale land-use changes likely affect sediment loading less than large-scale changes. Constructing retention ponds in association with large-scale tidal creek development activities, should also help mitigate excessive sediment loading to downstream coastal habitats. The developed watershed area of Howe Creek increased from 4.5% in 1959 to 70.0% in 2016, but MAR did not rapidly increase unlike the other sites in NHC. Furthermore, initial development of the Hewletts Creek watershed occurred within a Carolina Bay depression with a central retention pond, and that phase of development was not associated with an increase in MAR, unlike the subsequent development of other watershed areas (S2 Fig). Retention ponds likely interrupted sediment transport pathways to downstream areas in both Hewletts and Howe creeks and should be applied more widely in development plans for coastal communities.

Connectivity between creek watersheds and downstream estuaries

The stream power of tidal creeks is low because of their low slope, low discharge, and large backwater effects from lunar tides [67]. Despite a low sediment transport capacity, the connectivity of tidal creek watersheds to downstream bay head shorelines was high, using the time lag between when erosion of the landscape from when a MLCC initiated and when that sediment began to accumulate at the bay head shoreline as a proxy [3]. The timing of the MLCC was contemporaneous with increases in MAR near the bayhead shoreline at eight of the 11 tidal creeks, within the resolution of the remote-sensing data set (5-10 years). Land-use change is known to promote increases in sediment flux in low-relief areas [3, 32], but connectivity and the magnitude of the increase tends to be greater for smaller systems with higher relief [68, 24, 69]. Creek watersheds within coastal prism incised valleys should be more connected to downstream bayhead shorelines than tributary incised valleys given their generally larger drainage basins and higher relief; however, this was not resolved with our data set. Oyster Creek (Site 1) is within a tributary incised valley, has the lowest relief watershed (2.3 m), and like the creeks within coastal prism incised valleys, the increase in MAR at the bayhead shoreline of Oyster Creek corresponded immediately with the increase in cleared forest area from silviculture activity (Fig 6). The reach of the tidal creeks from headwater to mouth is short, only 5-15 km in length for the creeks in this study, and the proportion of the watersheds that experienced a change in land cover was high (>50%) for most of the creeks. The short length of the creeks and large percent change in land cover explains why these small watersheds are highly connected to downstream bay head shorelines and we see no correlations between connectivity and drainage basin size or relief. Whiskey Creek (Site 12) has the smallest drainage basin area and relief in NHC; however, it recorded similar trends in MAR as adjacent Hewletts Creek (Site 11), both accelerating with an

increase in development of the watersheds, despite Hewletts Creek having >three times greater drainage basin area and relief. In addition, the ~30% greater tidal range in NHC, as compared to CC, had little influence on connectivity. The MARs of Gales Creek, the only CC creek within a coastal prism incised valley accelerated with an increase in development after the MLCC, like the creeks in NHC (Fig 6.).

Average SAR pre and post MLCC

Sediment accumulation rates (SAR) provide an indicator of changes in bed elevation, and in coastal depocenters SAR typically match rates of relative sea-level rise over long timescales [42]. Half of the creek sites experienced a doubling or more in average SAR post MLCC, but all sites except Howe creek (site 9) showed an acceleration in SAR post-MLCC (Fig 7), despite the difference in type and percent land cover change between NHC and CC creeks. This widespread increase in average SARs can be attributed to increases in sediment load and increased accommodation through RSLR. There is a gradient in average 20th century rates of RSLR along the NC coast, increasing toward the north from 2.1 ± 0.5 mm/yr at Wilmington, near the NHC creeks to 3.5 ± 0.3 mm/yr at Tump Point, near CC creek sites [70]. Most sites recorded average SARs >3.5 mm yr^{-1} and up to ~8.4 mm yr^{-1} (Gales Creek, Site 6; Fig 7) post-MLCC, suggesting that these tidal creeks are infilling and getting shallower. Accommodation limits SAR and some sediment is likely bypassing and entering the adjacent estuary in CC creeks and the coastal ocean in NHC, especially during storm events, which could be contributing to higher MAR and SAR seen regionally in larger estuarine depocenters [15].

Fig 7. SARS Pre vs Post MLCC.
Average Pre and Post MLCC sediment accumulation rates compared to a 2:1 and a 1:1 line. Error bars represent average measurement error.

Conclusion

Tidal creek watersheds are small but abundant along passive margin coasts, worldwide, and formed within tributary and coastal prism incised valleys on the lower coastal plain. Despite variations in setting, watershed area and relief, and tidal range, tidal creek watersheds are directly connected to downstream bay head shorelines. Our data set cannot be used to explicitly identify sediment sources; however, MARs measured downstream, near the bay head shoreline, accelerated at the same time large changes in land cover occurred in the watershed at eight of eleven sites. This indicates sediment transport pathways in tidal creeks are more direct and responsive to land cover change than what their low relief and small watersheds would suggest. Much of the rapid increase in population of coastal areas and associated changes in land cover over the last 60 years occurred within tidal creek watersheds, and we documented a 10-15-fold increase in developed watershed area at four of the sites in NHC. Prior to MLCC, the average MAR at the sites were similarly low. At the end of the 20th century, the sites within coastal prism incised valleys, where development was the MLCC, showed higher average MARs than lower relief smaller tidal creeks within tributary incised valleys, where silviculture was the MLCC. The type of MLCC and/or watershed morphology influence the amount of sediment accumulating in down-stream coastal areas, and watersheds with small-scale changes in land cover or large changes in land cover with retention ponds showed little changes MAR at the tidal creek outlet. Most of the tidal creek bay head shoreline areas are accumulating sediment faster than the rate of RSLR; however, as RSLR continues to accelerate and these watersheds have little additional

space to accommodate future changes in land cover, this is likely a temporary state. Tidal creeks discharge directly into the flood plain of larger river systems, estuaries, or the coastal ocean and their contribution to coastal sediment budgets is largely ignored but could partly explain the increase that occurred after 1950 at many sites across North America.

REFERENCES

1. Barbier, E. B., Hacker, Sally D., Kennedy, Chris, Koch, Evamaria W., Stier, Adrian C., Silliman, Brian R. (2011). The value of estuarine and coastal ecosystem services. *Ecological Monographs, 81*(2), 169-193.

2. Grabowski, J. H., Brumbaugh, R. D., Conrad, R. F., Keeler, A. G., Opaluch, J. J., Peterson, C. H., Piehler, M. F., Powers, S. P., & Smyth, A. R. (2012). Economic Valuation of Ecosystem Services Provided by Oyster Reefs. *BioScience, 62*(10), 900-909. https://doi.org/10.1525/bio.2012.62.10.10

3. Mattheus, C. R., Rodriguez, A. B., & McKee, B. A. (2009). Direct connectivity between upstream and downstream promotes rapid response of lower coastal-plain rivers to land-use change. *Geophysical Research Letters, 36*(20). https://doi.org/10.1029/2009gl039995

4. Fagherazzi, S., Carniello, L., D'Alpaos, L., Defina, A. (2006) Critical bifurcation of shallow microtidal landforms in tidal flats an salt marshes. *PNAS, 103*(22). http://www.jstor.org/stable/30049243

5. Bromber, K. D., Bertness, M.D. (2005) Reconstructing New England salt marsh losses using historical maps. *Estuaries, 28*(6), 823-832.

6. Vermeer, M., Rahmstorf, S. (2009) Global sea level linked to global temperature. *PNAS, 106*(51). www.pnas.org/cgi/doi/10.1073/pnas.0907765106

7. Kirwan, M. L., Guntenspergen, G. R., D'Alpaos, A., Morris, J. T., Mudd, S. M., & Temmerman, S. (2010). Limits on the adaptability of coastal marshes to rising sea level. *Geophysical Research Letters, 37*(23), 1-5. https://doi.org/10.1029/2010GL045489

8. Ward, R. D., Friess, D. A., Day, R. H., & Mackenzie, R. A. (2016). Impacts of climate change on mangrove ecosystems: a region by region overview. *Ecosystem Health and Sustainability, 2*(4). https://doi.org/10.1002/ehs2.1211

9. Watson, E. B., Wigand, C., Davey, E. W., Andrews, H. M., Bishop, J., & Raposa, K. B. (2017). Wetland loss patterns and inundation-productivity relationships prognosticate widespread salt for southern New England. *Estuaries Coast, 40*(3), 662-681. https://doi.org/10.1007/s12237-016-0069-1

10. McLeod, E. & Salm, R.V. (2006). Managing mangroves for resilience to climate change. *IUCN*, Gland, Switzerland. 64pp.

11. Barbier, E.B., Koch, E.W., Silliman, B.R., Hacker, S.D., Wolancski, E., Primavera, J. (2008). Coastal ecosystem-based management with nonlinear ecological functions and values. *Science, 319*, 321. DOI: 10.1126/science.1150349.

12. Temmerman, S., Meire, P., Bouma, T. J., Herman, P. M., Ysebaert, T., & De Vriend, H. J. (2013). Ecosystem-based coastal defense in the face of global change. *Nature, 504*(7478), 79-83. https://doi.org/10.1038/nature12859

13. Möller, I., Kudella, M., Rupprecht, F., Spencer, T., Paul, M., van Wesenbeeck, B. K., et al., (2014). Wave attenuation over coastal salt marshes under storm surge conditions. *Nature Geoscience, 7*(10), 727-731. https://doi.org/10.1038/ngeo2251

14. National Oceanic and Atmospheric Administration. (2013). National coastal population report: Population trends from 1970-2020. NOAA, Department of Commerce.

15. Rodriguez, A. B., McKee, B. A., Miller, C. B., Bost, M. C., & Atencio, A. N. (2020). Coastal sedimentation across North America doubled in the 20(th) century despite river dams. *Nat Commun, 11*(1), 3249. https://doi.org/10.1038/s41467-020-16994-z

16. Miller, M. L. (1993). The rise of coastal and marine tourism. *Ocean & Coastal Management, 20*(3), 181-199. https://doi.org/10.1016/0964-5691(93)90066-8

17. Victor, S., Golbuu, Y., Wolanski, E., Richmond, R.H. (2004) Fine sediment trapping in two mangrove-fringed estuaries exposed to contrasting land-use intensity, Palau, Micronesia. *Wetlands Ecology and Management, 12*, 277-283.

18. Mattheus, C. R., Rodriguez, A. B., McKee, B. A., Currin, C. A. (2010). Impact of land-use change and hard structures on the evolution of fringing marsh shorelines. *Estuarine, Coastal and Shelf Science, 88*(3), 365-376. https://doi.org/10.1016/j.ecss.2010.04.016

19. Gunnell, J. R., Rodriguez, A. B., & McKee, B. A. (2013). How a marsh is built from the bottom up. https://doi.org/10.1130/G34582.1

20. Foley, J. A., Defries, R., Asner, G. P., Barford, C., Bonan, G., Carpenter, S. R., et al., (2005). Review: Global Consequences of Land Use. *8*, 570-574. https://doi.org/10.1126/science.1111772

21. James, L. A., & Lecce, S. A. (2013). 9.37 Impacts of Land-Use and Land-Cover Change on River Systems. In *Treatise on Geomorphology* (pp. 768-793). https://doi.org/10.1016/b978-0-12-374739-6.00264-5

22. Hupp, C. R., Schenk, E. R., Richter, J. M., Peet, R. K., & Townsend, P. A. (2009). Bank erosion along the dam-regulated lower Roanoke River, North Carolina. *Management and Restoration of Fluvial Systems with Broad Historical Changes and Human Impacts*. https://doi.org/10.1130/2009.2451(06)

23. Renfro, G.W. (1975). Use of erosion equations and sediment-delivery ratios for predicting sediment yield. *U.S. Department of Agriculture, S-40*, 33-45.

24. Milliman, J.D., Syvitski, J.P.M. (1992). Geomorphic/Tectonic Control of Sediment Discharge to the Ocean: The Importance of Small Mountainous Rivers. *The Journal of Geology, 100*(5), 525-544. https://www.jstor.org/stable/30068527

25. Syvitski, J. P. M., Milliman, J. D. (2007). Geology, Geography, and Humans Battle for Dominance over the Delivery of Fluvial Sediment to the Coastal Ocean. *The Journal of Geology, 115*(1), 1-19. https://doi.org/https://doi.org/10.1086/509246

26. McCarney-Castle, K., Voulgaris, G., & Kettner, A. J. (2010). Analysis of Fluvial Suspended Sediment Load Contribution through Anthropocene History to the South Atlantic Bight Coastal Zone, U.S.A. *The Journal of Geology, 118*(4), 399-416. https://doi.org/10.1086/652658

27. Weston, N. B. (2014). Declining Sediments and Rising Seas: an Unfortunate Convergence for Tidal Wetlands. *Estuaries and Coasts, 37*(1), 1-23. https://doi.org/10.1007/s12237-013-9654-8

28. Nienhuis, J. H., Ashton, A. D., Edmonds, D. A., Hoitink, A. J. F., Kettner, A. J., Rowland, J. C., et al., (2020). Global-scale human impact on delta morphology has led to net land area gain. *Nature, 577*(7791), 514-518. https://doi.org/10.1038/s41586-019-1905-9

29. Beighley, R.E., Melack, J.M., Dunne, T. (2003) Impacts of California's climatic regimes and coastal land use change on streamflow characteristics. *Journal of the American Water Resources Association,* 1419-1433.

30. Phillips, J.D. & Slattery, M.C., (2006). Sediment storage, sea level, and sediment delivery to the ocean by coastal plain rivers. *Progress in Physical Geography, 30*(4), 613-540. https://doi.org/10.1191/0309133306pp494ra

31. Kirwan, M. L., Murray, A. B., Donnelly, J. P., & Corbett, D. R. (2011). Rapid wetland expansion during European settlement and its implication for marsh survival under modern sediment delivery rates. *Geology, 39*(5), 507-510. https://doi.org/10.1130/g31789.1

32. Corbett, D. R., Walsh, J. P., & Zhao, Y. (2017). Impacts of Land-Use Change on Sedimentation in Tidal Creeks of North Carolina, USA. *Journal of Geography and Earth Sciences*. https://doi.org/10.15640/jges.v5n1a1

33. Darrow, E. S., Carmichael, R. H., Calci, K. R., & Burkhardt, W. (2017). Land-use related changes to sedimentary organic matter in tidal creeks of the northern Gulf of Mexico. *Limnology and Oceanography, 62*(2), 686-705. https://doi.org/10.1002/lno.10453

34. Schumm, S. A. (1993). Managing Mangroves for Resilience to Climate Change. *The Journal of Geology, 101*(2), 279-294. https://www.jstor.org/stable/30081152

35. Schumm, S. A., & Etheridge, F. G. (1994). Origin, Evolution and Morphology of Fluvial Valleys. *Incised Valley Systems: Origin and Sedimentary Sequenses, 51*, 11-27.

36. Greene, D.L., Rodriguez, A.B., Anderson, J.B. (2007) Seaward-Branching Coastal-Plain and Piedmont Incised-Valley Systems Through Multiple Sea-Level Cycles: Late Quaternary Examples from Mobile Bay and Mississippi Sound, U.S.A. *Journal of Sedimentary Research, 77*(2), 139-158. DOI: https://doi.org/10.2110/jsr.2007.016

37. Mattheus, C. R., & Rodriguez, A. B. (2014). Controls On Lower-Coastal-Plain Valley Morphology and Fill Architecture. *Journal of Sedimentary Research, 84*(4), 314-325. https://doi.org/10.2110/jsr.2014.30

38. Dalrymple, R. W., Zaitlin, B. A., & Boyd, R. (1992). Estuarine facies models; conceptual basis and stratigraphic implications. *Journal of Sedimentary Research, 62*(6), 1130-1146. https://doi.org/10.1306/D4267A69-2B26-11D7-8648000102C1865D

39. Mallin, M. A., & Lewitus, A. J. (2004). The importance of tidal creek ecosystems. *Journal of Experimental Marine Biology and Ecology, 298*(2), 145-149. https://doi.org/10.1016/S0022-0981(03)00356-3

40. Ensign, S., Currin, C., Piehler, M., & Tobias, C. (2017). A method for using shoreline morphology to predict suspended sediment concentration in tidal creeks. *Geomorphology, 276*, 280-288. https://doi.org/10.1016/j.geomorph.2016.09.036

41. Jervey, M. T., Wilgus, C. K., Hastings, B. S., Posamentier, H., Wagoner, J. V., Ross, C. A., et al., (1988). Quantitative Geological Modeling of Siliciclastic Rock Sequences and Their Seismic Expression. In *Sea-Level Changes: An Integrated Approach*, *42*. SEPM Society for Sedimentary Geology. https://doi.org/10.2110/pec.88.01.0047

42. Nichols, M. A. (1989). Sediment Accumulation Rates and Relative Sea-Level Rise in Lagoons. *Marine Geology, 88*, 209-219.

43. Riggs, S.R., Ames, D.V. (2003) Drowning the North Carolina coast: Sea-level rise and estuarine dynamics. *North Carolina Sea Grant,* ISBN 0-9747801-0-3.

44. Mallinson, D., Culver, S., Leorri, E., Mitra, S., Mulligan, R., & Riggs, S. (2018). Barrier Island and Estuary Co-evolution in Response to Holocene Climate and Sea-Level Change: Pamlico Sound and the Outer Banks Barrier Islands, North Carolina, USA. In *Barrier Dynamics and Response to Changing Climate* (pp. 91-120). https://doi.org/10.1007/978-3-319-68086-6_3

45. Ewen, C. R., Whyte, T.R., Davis Jr., R.P.S. (2011). The Archaeology of North Carolina: Three Archaeological Symposia. http://www.rla.unc.edu/NCAC/Publications/NCAC30/index.html

46. Prouty, W.F., 1952. Carolina bays and their origin. Bulletin of the Geological Society of America 63, 167–224.

47. NOAA OCM (National Oceanic and Atmospheric Administration, Office for Coastal Management), 2014, North Carolina Floodplain Mapping Program, https://coast.noaa.gov/dataviewer/#/lidar/search/where:ID=4954: accessed December 2017.

48. New Hanover County, 2015, Open Geospatial Data Portal: Watersheds, http://geo.nhcgov.com/gis/rest/services/Layers/Watersheds/MapServer/0, accessed June 2016.

49. NOAA OCM (National Oceanic and Atmospheric Administration, Office for Coastal Management), 2016, Coastal Change Analysis Program (C-CAP) Regional Land Cover,

www.coast.noaa.gov/ccapftp: accessed December 2017.

50. Simms, A.R., Rodriguez, A.B. Anderson, J.B. (2018) Bayhead deltas and shorelines: Insights from modern and ancient examples. *Sedimentary Geology, 274*, 17-35.

51. Heiri, O., Lotter, A.F., Lemcke, G. (2001) Loss on ignition as a method for estimating organic and carbonate content in sediments: reproducibility and comparability of results. *Journal of Paleolimnology, 25*, 101-110.

52. Flynn, W.W. (1968) The determination of low levels of Polonium-210 in environmental materials. *Analytica Chimica Acta., 43,* 221-227.

53. El-Daoushy, F., Olsson, K., Garcia-Tenorio, R. (1991). Accuracies in PO-210 determination for lead-210 dating. *Hydrobiologia 214*, 43-52.

54. Matthews, K. M., Kim, C.-k., & Martin, P. (2007). Determination of Po in environmental materials : A review of analytical methodology. *65*, 267-279. https://doi.org/10.1016/j.apradiso.2006.09.005

55. de Vleeschouwer, F., Sikorski, J., & Fagel, N. (2010). Development of Lead-210 Measurement in Peat Using Polonium Extraction. A Procedural Comparison. *Geochr, 36*(-1), 1-8. https://doi.org/10.2478/v10003-010-0013-5

56. Sanchez-Cabeza, J. A., & Ruiz-Fernández, A. C. (2012). 210Pb sediment radiochronology: An integrated formulation and classification of dating models. *Geochimica et Cosmochimica Acta, 82*, 183-200. https://doi.org/10.1016/j.gca.2010.12.024

57. Appleby, P. G., & Oldfield, F. (1978). The calculation of lead-210 dates assuming a constant rate of supply of unsupported 210-Pb to the sediments. wird eine Methode zur Berechnung des Sedimentalters beschrieben , die auf 210pb. *Catena, 5*, 1-8.

58. Wells, J. T., Kim, S. (1989). Sedimentation in the Albemarle-Pamlico lagoonal system: synthesis and hypothesis. *Marine Geology, 88*, 263-284.

59. Cooper, S.R., McGlothlin, S.K., Madritch, M., Jones, D.L. (2004) Paleoecological evidence of human impacts on the Neuse and Pamlico estuaries of North Carolina, USA. *Estuaries, 27(*4), 617-633.

60. Miller, C. B., Rodriguez, A. B., & Bost, M. C. (2021). Sea-level rise, localized subsidence, and increased storminess promote saltmarsh transgression across low-gradient upland areas. *Quaternary Science Reviews, 265*. https://doi.org/10.1016/j.quascirev.2021.107000

61. Torres, R., Mwamba, M.J., Goni, M.A. (2003) Properties of intertidal marsh sediment mobilized by rainfall. *Limnology and Oceanography, 48* (3), 1245-1253.

62. Paerl., H.W., Hall, N.S., Hounshell, A.G., Rossignol, K.L., Barnard, M.A., Luettich Jr., R.A. et al., (2020) Recent increases of rainfall and flooding from tropical cyclones (TCs) in North Carolina (USA): implications for organic matter and nutrient cycling in coastal watersheds.

Biogeochemistry, 150, 197-216. https://link.springer.com/article/10.1007/s10533-020-00693-4

63. Knutson, T.R., McBride, J. L., Chan, J., Emanuel, K., Holland, G., Landsea, C., et al., (2010) Tropical cyclones and climate change. *Nature Geoscience, 3*. DOI: 10.1038/NGEO779

64. Gambrell, R. P. (1994). Trace and Toxic Metals in Wetlands-A Review. *Journal of Environmental Quality, 23*, 883-891.

65. Sanger, D. M., Holland, A.F., Scott, G.I. (1999). Tidal Creek and Salt Marsh Sediments in South Carolina Coastal Estuaries: I. Distribution of Trace Metals. *Archives of Environmental Contamination and Toxicology, 37*, 445-457.

66. NCAC (North Carolina Administrative Code). (2007) rule 15A NCAC 03N.0101. http://reports.oah.state.nc.us/ncac/title%2015a%20-%20environmental%20quality/chapter%2003%20-%20marine%20fisheries/subchapter%20n/subchapter%20n%20rules.pdf

67. Slattery, M.C., Phillips, J.D. (2011) Controls on sediment delivery in coastal plain rivers. *Journal of Environmental Management, 92*(2), 284-289. DOI: 10.1016/jjenvman.2009.08.022

68. Syvitski, J. P. M. (1978). Sediment fluxes and rates of sedimentation. In *Sedimentology* (pp. 980-992). https://doi.org/10.1007/3-540-31079-7_180

69. Douglas, I. (1993) Chp. 13 Sediment transfer and siltation. In: The Earth as transformed by human action: Global and regional changes in the Biosphere over the past 300 years. *Press syndicate of the University of Cambridge*

70. Kopp, R.E., Horton, B.P., Kemp, A.C., Tebaldi, C. (2015) Past and future sea-level rise along the coast of North Carolina, USA. *Climatic Change.* DOI: 10.1007/s10584-015-1451-x

Figure 1

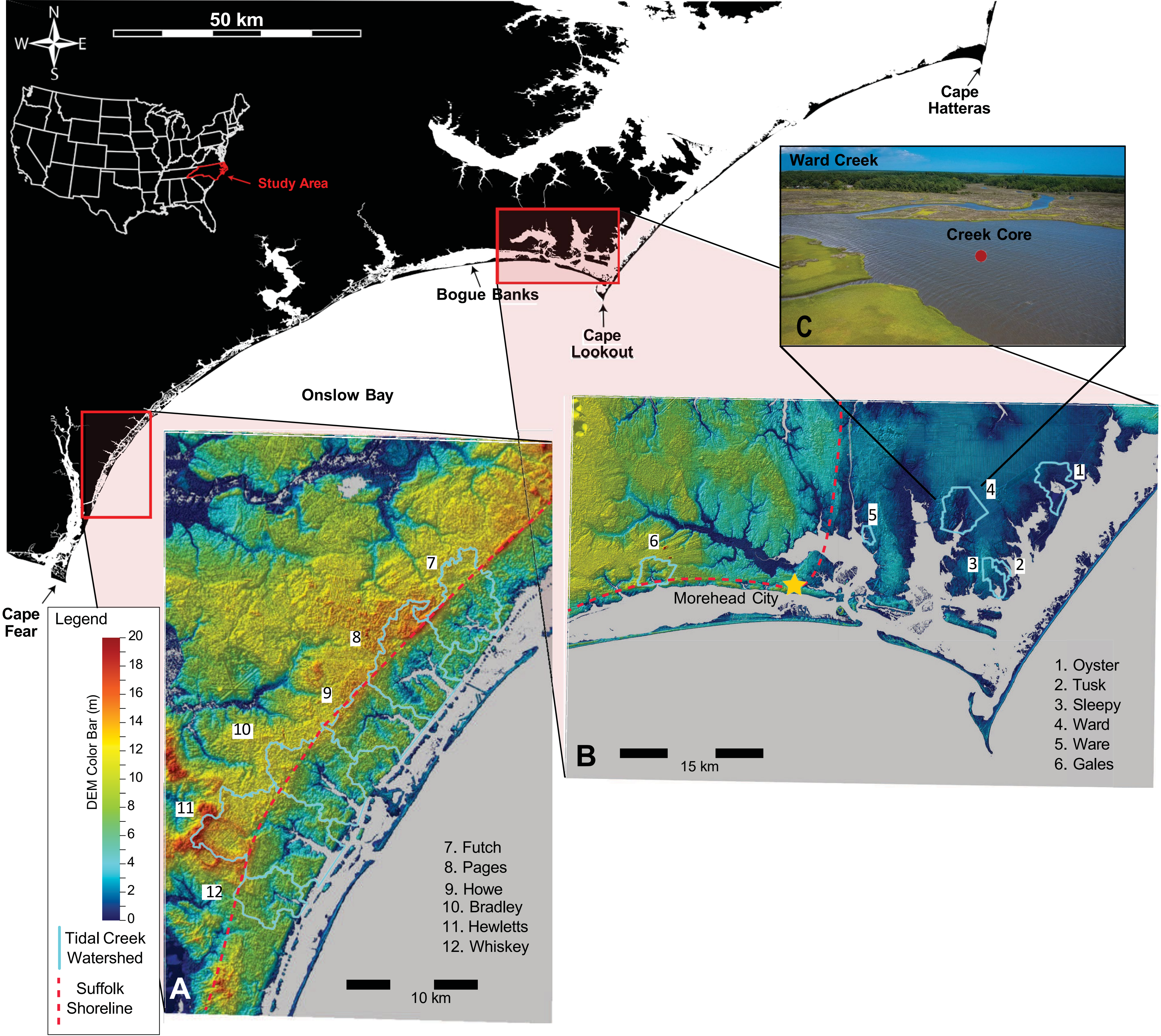

Click here to access/download;Figure;Fig 1.eps

Figure 2

Click here to access/download;Figure;Fig 2.eps

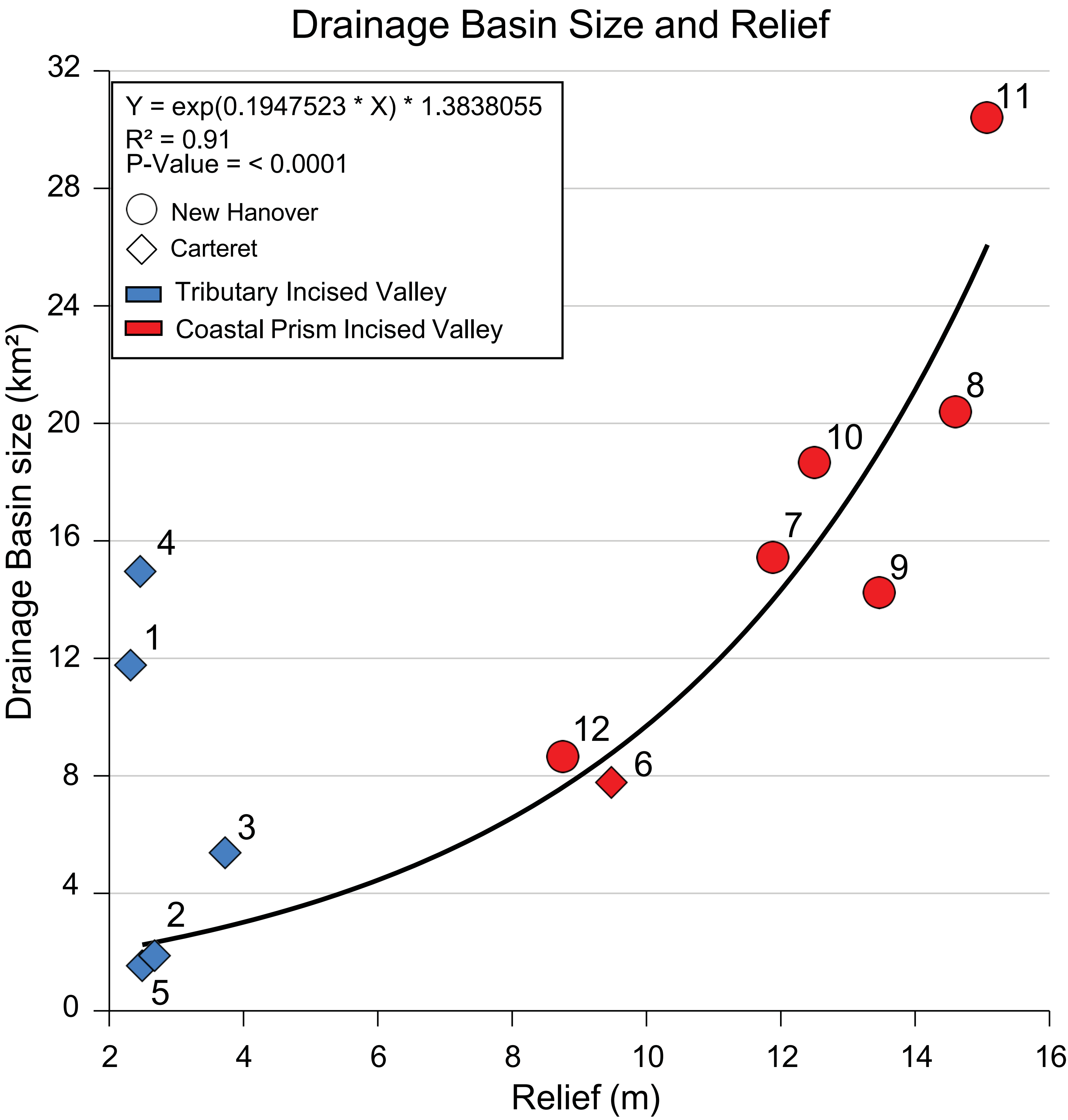

Figure 3

Click here to access/download;Figure;Fig 3.eps

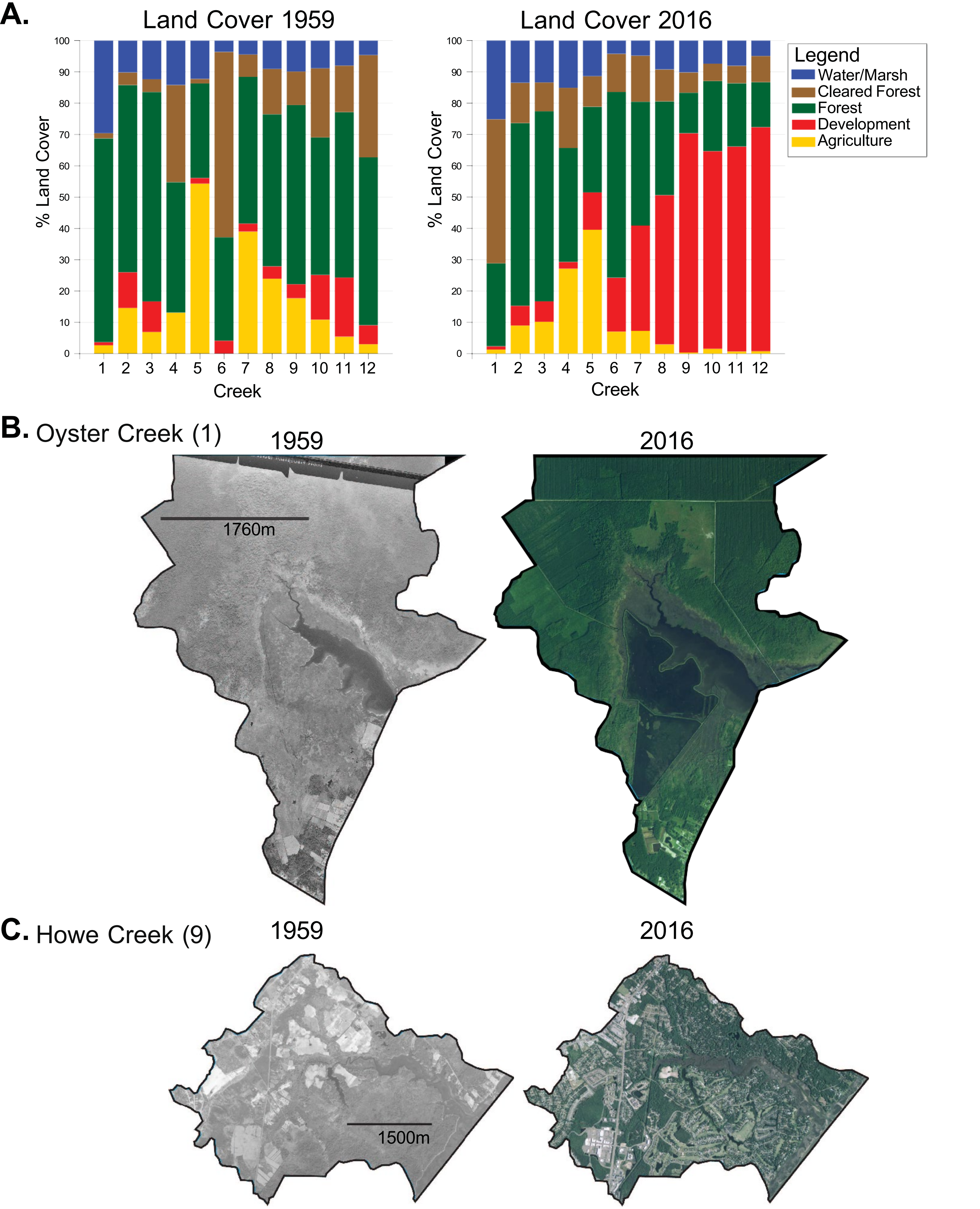

Figure 4

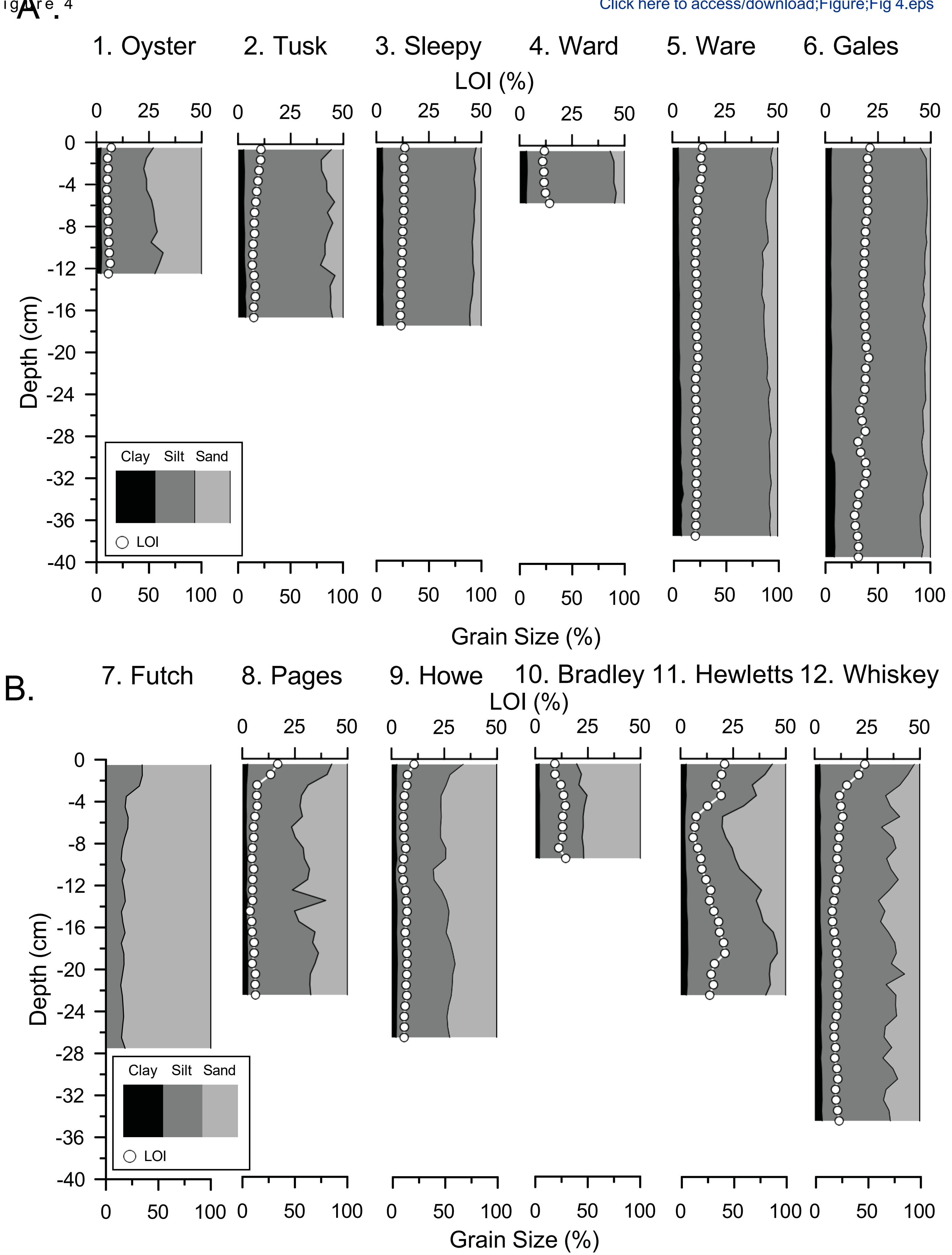
A.
1. Oyster
2. Tusk
3. Sleepy
4. Ward
5. Ware
6. Gales
LOI (%)
0 25 50
Depth (cm)
0 -4 -8 -12 -16 -20 -24 -28 -32 -36 -40
Clay Silt Sand
LOI
0 50 100
Grain Size (%)
B.
7. Futch
8. Pages
9. Howe
10. Bradley
11. Hewletts
12. Whiskey
LOI (%)
0 25 50
Depth (cm)
0 -4 -8 -12 -16 -20 -24 -28 -32 -36 -40
Clay Silt Sand
LOI
0 50 100
Grain Size (%)

Figure 5

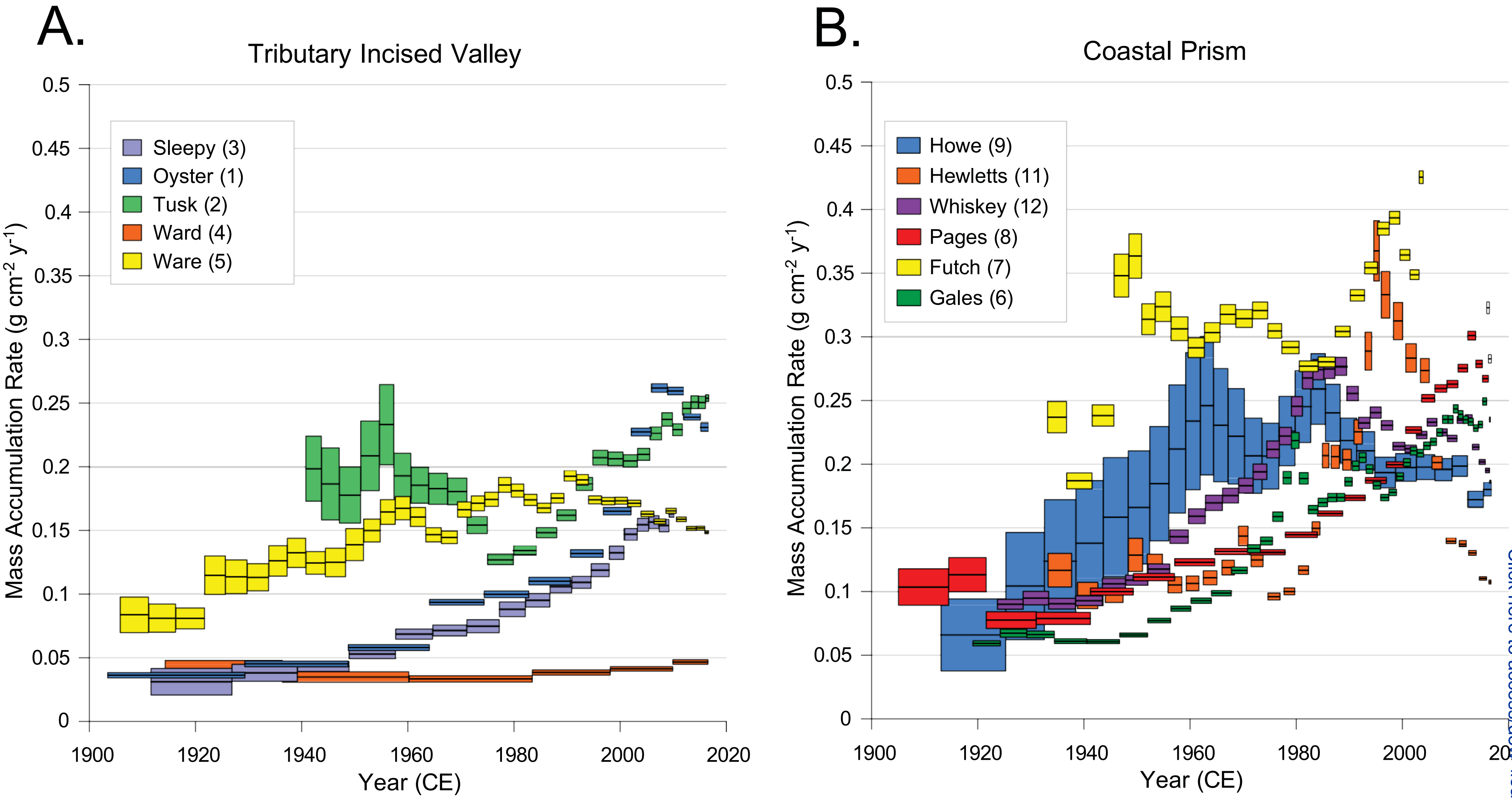

Click here to access/download;Figure;Fig 5.eps

Figure 6

Click here to access/download;Figure;Fig 6.eps

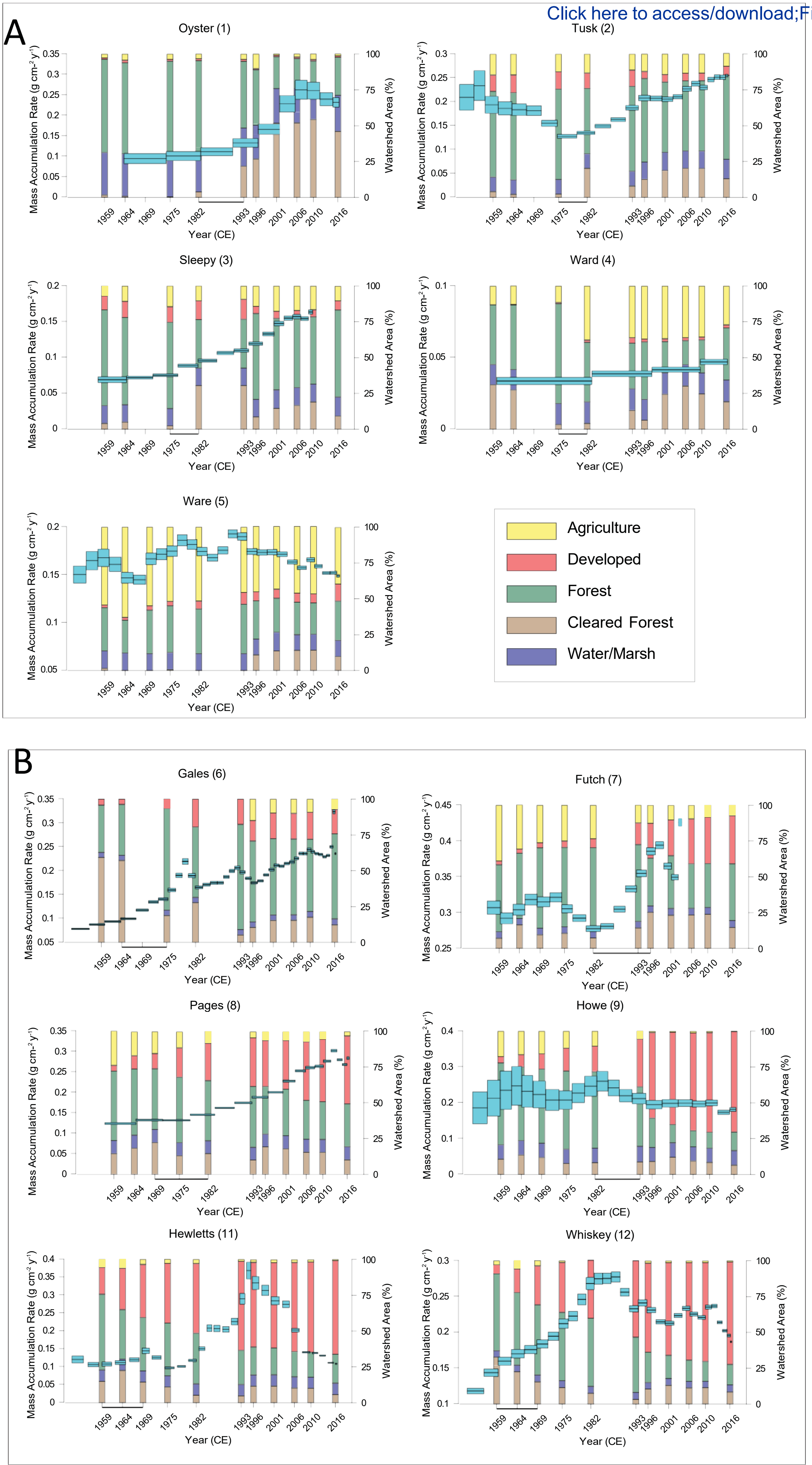

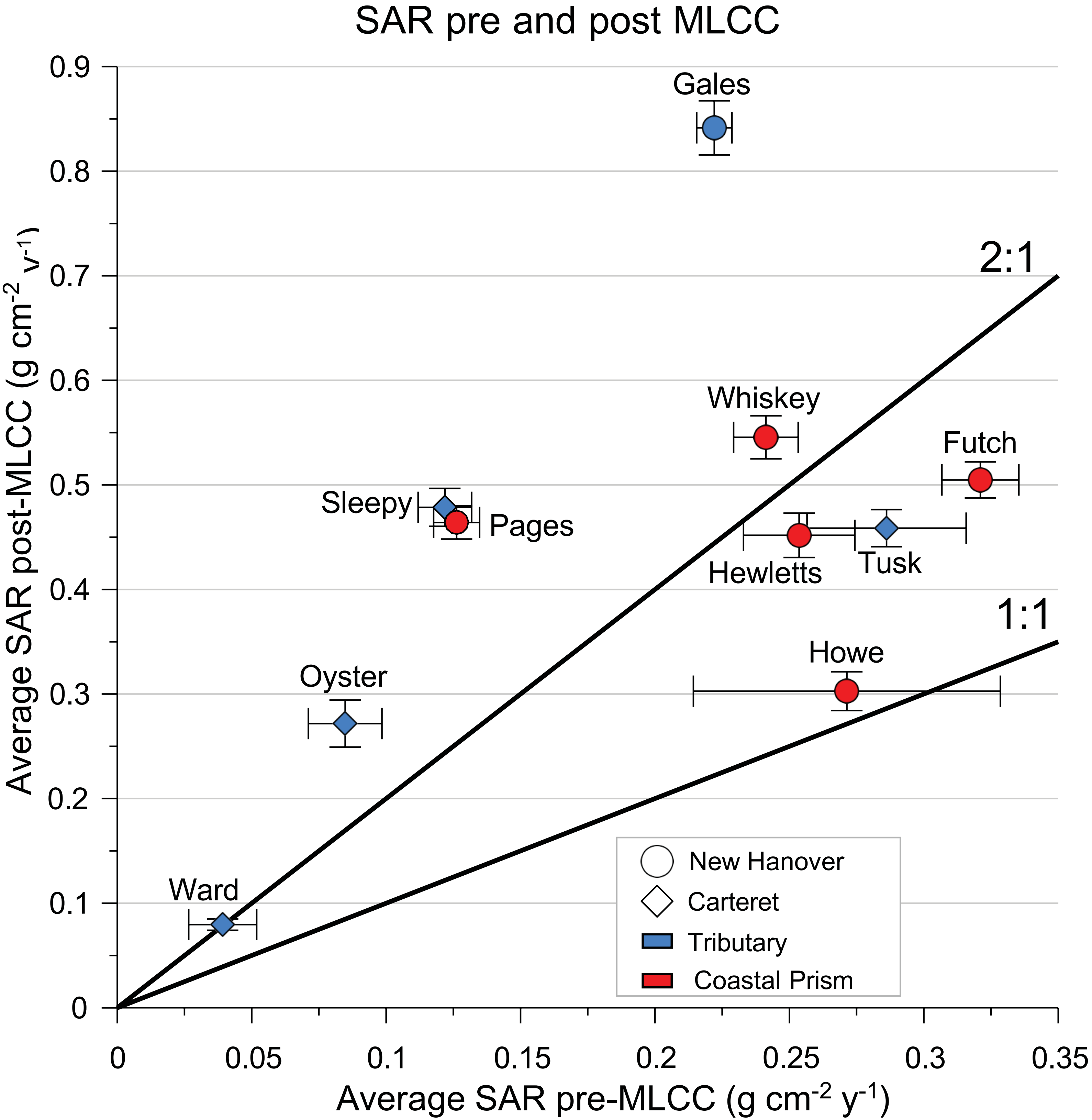

Click here to access/download;Figure;Fig7.eps

Click here to access/download;Figure;S1 Fig.eps

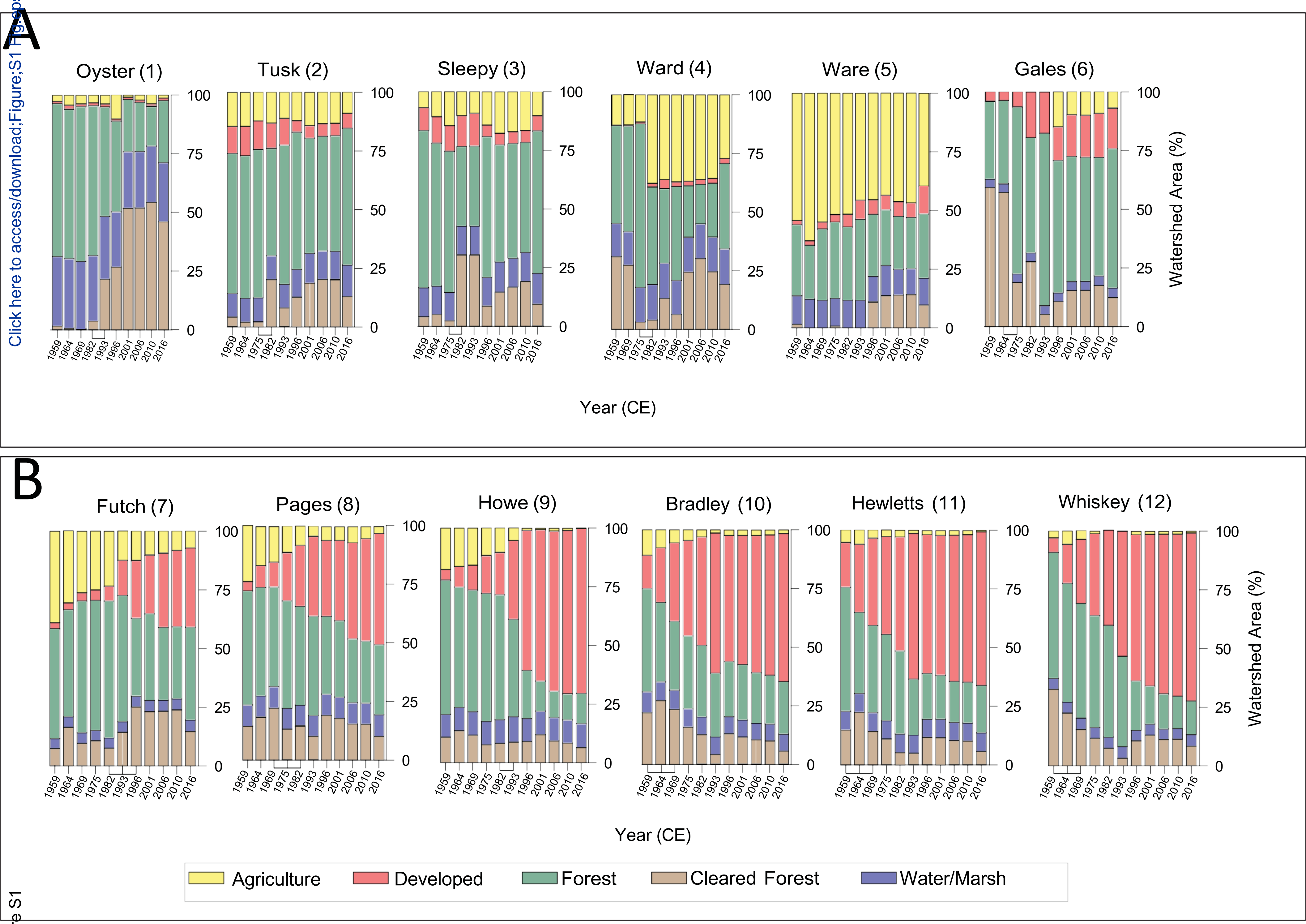

Figure S1

Figure S2

Click here to access/download;Figure;S2 Fig.eps

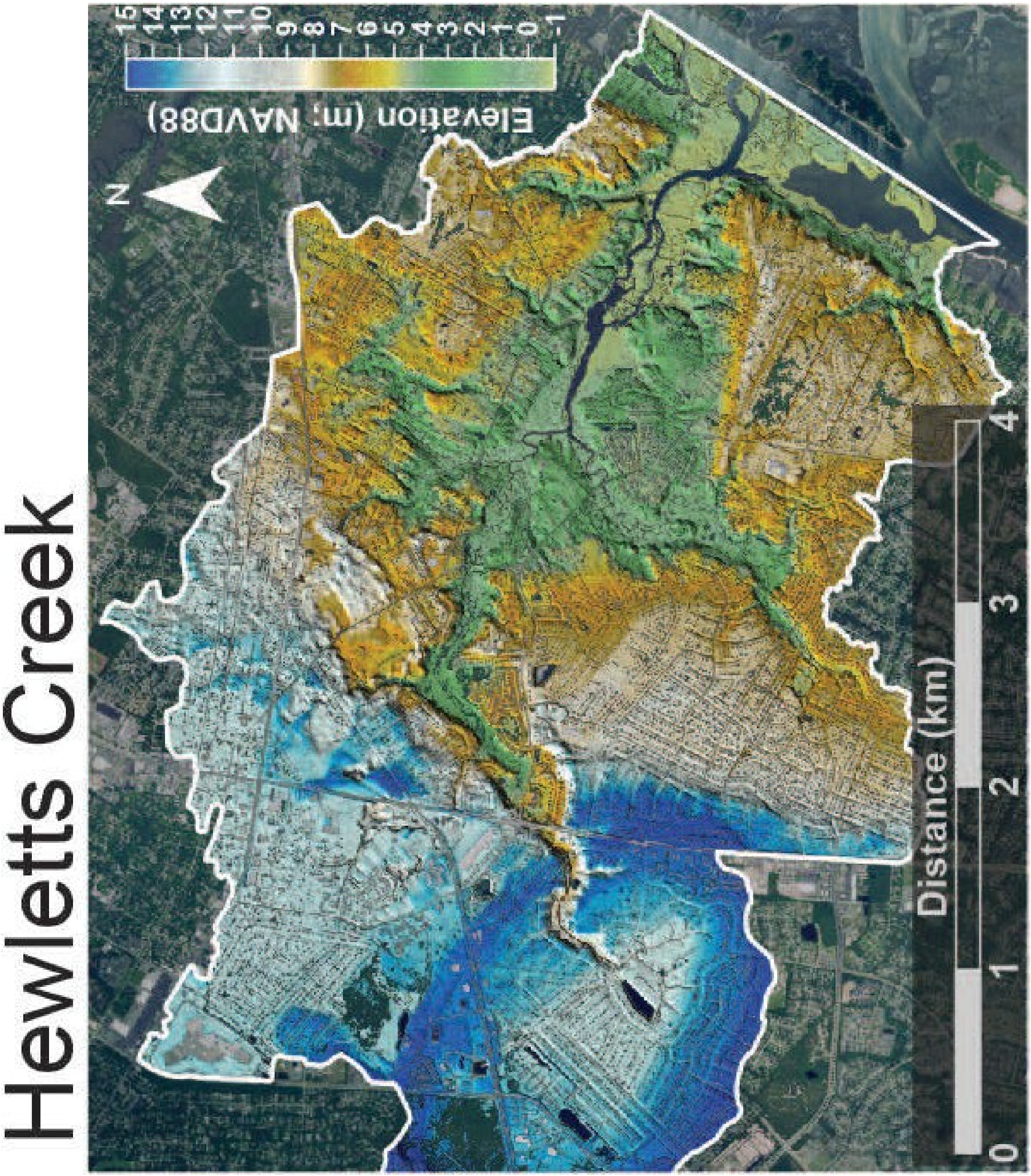

Click here to access/download
Supporting Information
Bost_Supporting Information.xlsx
