# Supplementary material for: Anthropogenic impacts on tidal creek sedimentation since 1900
Source: PLoS One. 2023 Jan 18;18(1):e0280490. doi: 10.1371/journal.pone.0280490 (PMC9847910; doi:10.1371/journal.pone.0280490)
Supplement: Attachment — Submitted filename: PONE-D-22-15917_R1_Response Letter.docx [file pone.0280490.s005.docx]

PONE-D-22-15917

Thank you all (Reviewers 1 & 2 as well as the editor) for taking the time to review this manuscript for publication at PLOS ONE. Your comments are appreciated and were very helpful in bettering this manuscript. Following are the comments from reviewers with my responses in **bold font.**

Anthropogenic impacts on tidal creek sedimentation since 1900

^1,2,3^Molly C. Bost, ^1^Charles D. Deaton, ^1,4^Antonio B. Rodriguez, ^2^Brent A. McKee, ^1,4^F. Joel Fodrie, ^1^Carson, B. Miller

1. *Institute of Marine Sciences, University of North Carolina at Chapel Hill, 3431 Arendell St., Morehead City, NC 28557, USA.*
2. *National Oceanic and Atmospheric Administration, National Centers for Coastal Ocean Science, Beaufort, NC, United States*
3. CSS-Inc., Fairfax, VA, United States
4. *Department of Earth, Marine and Environmental Sciences, University of North Carolina at Chapel Hill, Chapel Hill, NC 27599-3300, USA.*

Reviewer #1: There are some interesting observations in this paper and its is a useful case study of small watershed and the implications of land cover changes. However, those findings are masked by an extremely complex typology of the landscape which is very difficult to follow, not well explained, and seems to come out of the blue. The paper really needs a better set up with some clear research questions (grounded in this typology is appropriate) and much more on the regional setting. There are a large number of very general statements and its not clear if these are meant to be global observations or of regional/local interest only. I would focus the paper much more on the land cover and sediment with only enough coastal plain geomorphology to understand what is going on. For a global reach journal context for NC will be important. The attached marked up copy includes some specific comments that should be useful in reframing

**We appreciated this feedback and have pared down the introduction significantly. Additionally, we removed the background section and included the ideas we deemed necessary in the introduction. The text is more streamlined now in the introduction and focuses primarily on sedimentation, land-use change, and introducing tidal creeks.**

**Comments made on the reviewed manuscript and pasted them below to address them here:**

Line 61: They don't really drown. The plants can't survive as the level of inundation changes the soil chemistry. Use some thing more generic like 'saltmarsh and mangrove transition to open water due to excessive inundation associated with sea-level rise'

**Line 64-66: This has been addressed and the reviewer’s suggestion has been incorporated into the text.**

Line 69: This is a very dated citation for something that may be on the increase

**Lines 80-83: This portion of the introduction has been reworked and this particular sentence regarding coastal tourism was deemed redundant and removed.**

Line 74: I think you are overstating this - the citations are about changes in sediment delivery not storm protection. Unless you really want to show the storm protection function I'd suggest you focus on the habitat transitions. They can be important in and of them selves (due to other ecosystem services) without tying everything to 'protection'

**Through the reworking of the introduction, this line has been removed and revised throughout the introduction.**

Line 89: I think you need to parse these ideas out a bit more. By shoreline you mean open coast? But sediment only gets to the open coast from the river if it passed through the estuary. All rivers have estuaries of one type or another. What do you mean by coastal zone - does that include the estuary. I think you know what you mean here but it’s a bit confusing in the current text. And how do 'drowned river mouths' come into this.

**Line 83-89: This section has been addressed and reworked to be more specific and some of the word choices the reviewer took issue with have now been removed.**

Line 95: What do you mean by this - sediment exchanges?

**Line 108-109: The reference to connectivity has been made clearer.**

Line 97: Topographic?

**Line 110: This change has been made.**

Line 101-104: Move the definition of size up

**Line 86-93: The definition of tidal creeks including size is now earlier in the introduction.**

Line 107: We need more information on this - how little, small etc

**Line 154: relief and area numbers have been included in the text now to give a better understanding of how small these systems are.**

Line 121: In your area - not always though

**Line 157: This statement now directly relates to tidal creeks specifically.**

Line 112: What is the time period for the analysis, frequency of data

**Table 1. Land-cover datasets. This table offers specifics for each year there was available data digitized for land cover. This analysis ranges from 5-10 year temporal resolution.**

Line 114: Its unclear how documenting the association provides guidance

**Line 160-164:** **This work will inform managers like those with the Erosion and Sediment Control Commission of North Carolina about the effectiveness of sediment control measures in the past in order to move forward with conservation practices. This sentence now includes that entity.**

Line 116: I think you need to do a better introduction to these management practices - how do you know if they have occurred during the period of analysis, as well as land cover

**Line 160-165: This section has been pared down to be more concise.**

Line 119: I think we need some research questions of hypotheses to tell us why this background information is relevant. The move to tidal creeks comes out of the blue here. Why is the Background separate from the Introduction?

**Line 136: The objective is now earlier in the text and more clearly related to the introduction text.**

Line 121: If you are talking about a specific place then it should be clear

**Line 94-107: This has been addressed with the removal of the background and its incorporation into the introduction.**

126: What is the difference?

**Line 86-69; 94-107: The difference between estuaries and tidal creeks has been made clearer.**

Line 128: So these are distinct from estuaries because there is no freshwater input

**Line 86-69; 94-107: Correct. This section has been rearranged.**

Line 131: How? (related to excluding wetland channels)

**This issue is no longer relevant given the new text. This has been removed and reworked within the introduction.**

Line 132: So why are they not estuaries?

Line 132: This is extremely confusing - and it is totally unclear why it is relevant. Your introduction should have included these topics so we know why they are relevant.

**The introduction/background section have been modified to be clearer.**

Line 141: You haven't actually talked about the study area yet

**Line 189: Fig 1 has been moved into the site selection section of the methods section.**

Line 225: How were the years selected

**Line 212: This is now addressed in the text as “based on imagery availability”**

Line 260: This would need to be annotated to indicate the characteristics of the location

**Line 248: Fig. 1C has been modified as suggested.**

Line 260: The picture shows the location is in water. Explain the coring method, how it was extracted, wall thickness, diameter and how compaction was taken into account.

**Line 248: This has been addressed with more information about the coring method.**

Line 347: Where is the data? Shouldn't you refer to it?

**Line 345: This has been fixed and Fig 4 is now referenced.**

Line 419: How? Related to “There is no hydrological connectivity between the NHC tidal creek sites and the nearby Cape Fear River, but sediment loading in the Neuse and the Newport rivers could have influenced sedimentation in the CC tidal creeks.”

**Line 467: This has been addressed by the inclusion of “as they do not share a watershed”**

Line 510: Unless you have measured this it seems speculative

**This issue has been resolved by the removal of this sentence entirely.**

Line 518: Why not avoid the sediment moving away from the development site rather than retaining it offsite.

**Line 495-510: This has been addressed more thoroughly.**

Line 526: I think you need to consider the array of approaches before you focus on this one.

**Line 495-510: This has been addressed. We focused primarily on discussion of the retention pond approach because two of our sites were greatly impacted by the presence of man-made retention ponds (Howe, site 9) and a Carolina Bay depression which arguably functions in the watershed as a large retention pond (Hewletts, Site 11).**

Line 579: These types of global statements are not real conclusions from your study

**Line 577: That’s true. This has been addressed and global statements are now avoided in the revised conclusion.**

Line 587: Focus on your study and findings

**Noted. This has been removed.**

Reviewer #2: This paper quantifies sediment accumulation rates in tidal creeks before and after 1950, identifies periods of greatest land cover change, and relates the changes in sedimentation to land use change in the creek watersheds. The results are valuable to better understand changes in sedimentation processes (in the light of coastal wetlands facing sea-level rise), and for coastal zone managers to avoid potentially undesired habitat transitions.

However, I have three main issues with the analysis and reporting of the results in this study.
First, the authors state that the timing of the increase in mass accumulation rates after 1959 corresponded with the major land cover change in 8 out of 12 creek sites. I understand that relating changes in mass accumulation rates with changes in land cover in a quantitative way is difficult because of different temporal resolutions (as the authors state in L444 – 447). However, how exactly do you define the time where mass accumulation rates start to increase? This is not so easy because in some cases you could argue that the increasing trend was already there before the major land cover shift (for instance, in creek 12 in Figure 6, or creek 11 where the increase seems to start later, around 1982, as is also stated in L462).

**We have put together a quantifiable way to discern when MAR accelerated. Similar to the defined MLCC, we chose to look only at changes between MARs beginning in 1959 (the start of the land-cover record). When the first acceleration of ≥10% occurred, we identified the timing and were able to align that within the MLCC for eight of the creek sites that had both a change in MAR through time and a MLCC. This excluded Sites 10, 4, 5, and 9 because site 10 had a mixed sedimentary record that could not be resolved, site 4 experienced no change in MAR throughout the record (within error), the watershed for site 5 did not have a defined MLCC, and Site 9 had no change in MAR throughout the record.**

**As the reviewer mentions, I already state why we suspect the MLCC was delayed in the case of Creek 11. In Creek 12, we suspect land-use may have already seen a shift (likely in cleared forest before 1959) which was outside the reach of our imagery. This may have caused MAR to start increasing before the increase in development area. Regardless, during the decade where the MLCC occurred, an increase in MAR is apparent until ~1982 when it begins to level off.**

Is it possible to identify the trends and shifts in mass accumulation rates in a more quantitative way? For instance, piecewise regression could be used to show in a more objective way that the data follow different trends over time and identify that breakpoint.

**We hesitate to put regressions through any of the MAR data because too many seemingly arbitrary choices would have to be made. We would be forced to use the midpoint date and the midpoint MAR which ignores much of the information provided by that point and may give misleading results.**

**We’ve now included a criterion for identifying this trend. Lines 273-278; 396-418.**

Second, the authors only measured sedimentation rates at the bottom of creeks, but the resilience of salt marshes would largely depend on the deposition of sediment on the salt marsh platform. How would these results on the filling-up of creeks translate to sediment transport on the vegetated platform? In the end, salt marsh platforms need to accrete to prevent drowning with sea-level rise.

**This is correct, we only measured sedimentation for this study at 12 creek bottom sites. The coring sites are all close to salt marsh (within ~25 m) and, considering the creek bed and adjacent salt marsh have the same allogenic sediment supply, our results are relevant to adjacent habitats. Future work will focus on those adjacent salt marshes.**

**The reviewer is correct that the resilience of salt marshes depends on sedimentation on the marsh platform and we allude to this as being a primary reason that sedimentation in coastal areas is important to study and manage for. However, the purpose of our study was to really compare local, coastal watershed LUC with any changes in sedimentation rates on the creek bed to assess connectivity as many studies have done before with larger riverine and estuarine systems. While studying the marsh would be valuable, it was outside the scope of this particular study.**

Finally, I think the manuscript structure should be improved. The Discussion section is mixed with a description of results and reference to figures that are introduced there for the first time. Figure 6 and 7 are only mentioned/described in the discussion: as far as I know, this is not very common (unless it’s a conceptual figure). I would first expect that all the main findings are presented in the results section (e.g., those reported in paragraph ‘Average SAR pre and post MLCC’).

**Thank you for this input. We agree and have reworked the discussion and results sections immensely. Specifically, we have moved Figures 6 and 7 to the results section.**

Specific comments

L21-22: I’m not sure about the phrasing “Small coastal watersheds (tidal creeks)”: the watershed is the area drained by the tidal channel, but not the channel itself. Please rephrase.

**Line 25-27: This has been rephrased.**

L40: this sentence refers to ‘one site’ and ‘another site’, but it is unclear if it refers to a creek or one of the two main regions that you studied. I suggest making it more specific.

**Line 43: This has been changed to be clearer.**

L211: What is the horizontal resolution of the LiDAR data? Is it the same for both sites?

**Line 196-201: This information has been clarified.**

Line 367: Figure 4 legend: please indicate the difference between A and B plots in the legend: CC creek sites in A, and NHC creek sites in B?

**Line 362: This has been added.**

Line 373- 383: Figure 5: maybe I missed this in the text, but can you explain what is causing this decline over the last 5 years or so?
**Original text Line 480-487; new text 492**

**“It is likely that surface sediment was eroded from the watershed and transported to the creeks during the construction phase of development, which included clearcutting forest, and once developed there was little sediment available that could be mobilized and exported to the tidal creeks.**

L507: what is meant by structured habitats?”

**Structured habitats like fringing salt marshes, salt marsh islands, oyster reefs, seagrass beds, and mangroves. This has been revised throughout the manuscript.**

L535 and L585: is it 11 or 12 creeks in total? Creek 10 is sometimes missing in the figures.

**The manuscript has been changed to address this**. **Now lines 392-395; 403-407; 553-555**

L562: Fig should be Fig.

**The format has been revised throughout the text to align with the PLOSONE requirements.**

L583-585: that sounds like an overstatement, because the time of MAR acceleration is not quantified in an objective way.

**We have now incorporated a more quantitative method to assess when an acceleration in MAR occurred that supports this statement.**
